# Genome-Wide Association Study of Cryptosporidiosis in Infants Implicates *PRKCA*

**DOI:** 10.1128/mBio.03343-19

**Published:** 2020-02-04

**Authors:** Genevieve L. Wojcik, Poonum Korpe, Chelsea Marie, Alexander J. Mentzer, Tommy Carstensen, Josyf Mychaleckyj, Beth D. Kirkpatrick, Stephen S. Rich, Patrick Concannon, A. S. G. Faruque, Rashidul Haque, William A. Petri, Priya Duggal

**Affiliations:** a Department of Biomedical Data Science, Stanford University School of Medicine, Stanford, California, USA; b Department of Epidemiology, Johns Hopkins Bloomberg School of Public Health, Baltimore, Maryland, USA; c Department of Medicine, Infectious Diseases and International Health, University of Virginia School of Medicine, Charlottesville, Virginia, USA; d Wellcome Trust Sanger Institute, University of Oxford, Oxford, United Kingdom; e Big Data Institute, Li Ka Shing Centre for Health Information and Discovery, University of Oxford, Oxford, United Kingdom; f Department of Medicine, University of Cambridge, Cambridge, United Kingdom; g Center for Public Health Genomics, Department of Public Health Sciences, University of Virginia, Charlottesville, Virginia, USA; h University of Vermont College of Medicine and Vaccine Testing Center, Burlington, Vermont, USA; i Genetics Institute and Department of Pathology, Immunology and Laboratory Medicine, University of Florida, Gainesville, Florida, USA; j International Centre for Diarrhoeal Disease Research, Bangladesh, Dhaka, Bangladesh; Harvard T.H. Chan School of Public Health

**Keywords:** *Cryptosporidium*, genetics, genome analysis

## Abstract

Diarrhea is a major cause of both morbidity and mortality worldwide, especially among young children. Cryptosporidiosis is a leading cause of diarrhea in children, particularly in South Asia and sub-Saharan Africa, where it is responsible for over 200,000 deaths per year. Beyond the initial clinical presentation of diarrhea, it is associated with long-term sequelae such as malnutrition and neurocognitive developmental deficits. Risk factors include poverty and overcrowding, and yet not all children with these risk factors and exposure are infected, nor do all infected children develop symptomatic disease. One potential risk factor to explain these differences is their human genome. To identify genetic variants associated with symptomatic cryptosporidiosis, we conducted a genome-wide association study (GWAS) examining 6.5 million single nucleotide polymorphisms (SNPs) in 873 children from three independent cohorts in Dhaka, Bangladesh, namely, the Dhaka Birth Cohort (DBC), the Performance of Rotavirus and Oral Polio Vaccines in Developing Countries (PROVIDE) study, and the Cryptosporidiosis Birth Cohort (CBC). Associations were estimated separately for each cohort under an additive model, adjusting for length-for-age Z-score at 12 months of age, the first two principal components to account for population substructure, and genotyping batch. The strongest meta-analytic association was with rs58296998 (*P* = 3.73 × 10^−8^), an intronic SNP and expression quantitative trait locus (eQTL) of protein kinase C alpha (*PRKCA*). Each additional risk allele conferred 2.4 times the odds of *Cryptosporidium*-associated diarrhea in the first year of life. This genetic association suggests a role for protein kinase C alpha in pediatric cryptosporidiosis and warrants further investigation.

## Abstract (precis)

Globally, diarrhea remains one of the major causes of pediatric morbidity and mortality. The initial symptoms of diarrhea can often lead to long-term consequences for the health of young children, such as malnutrition and neurocognitive developmental deficits. Despite many children having similar exposures to infectious causes of diarrhea, not all develop symptomatic disease, indicating a possible role for human genetic variation. Here, we conducted a genetic study of susceptibility to symptomatic disease associated with *Cryptosporidium* infection (a leading cause of diarrhea) in three independent cohorts of infants from Dhaka, Bangladesh. We identified a genetic variant within protein kinase C alpha (*PRKCA*) associated with higher risk of cryptosporidiosis in the first year of life. These results indicate a role for human genetics in susceptibility to cryptosporidiosis and warrant further research to elucidate the mechanism.

## INTRODUCTION

Cryptosporidiosis is a leading cause of diarrhea and is estimated to be responsible for greater than 200,000 deaths in young children in South Asia and sub-Saharan Africa each year (1). Beyond the immediate infection, cryptosporidiosis is also associated with long-term sequelae, including malnutrition and neurocognitive developmental deficits (2–5). The majority of human infections are caused by the Cryptosporidium hominis, C. meleagridis, and C. parvum species (4, 6, 7). As cryptosporidiosis is transmitted fecal-orally, contact with any reservoir with possible fecal contamination could serve as a point of transmission. In the developed world, cryptosporidia represent an important cause of diarrhea in individuals living with HIV and are the most common pathogens causing waterborne outbreaks (7).

In regions of endemicity, cryptosporidiosis mostly impacts young children, and risk factors for infection include poverty and overcrowding (4, 8–10). Livestock serve as an environmental reservoir for C. parvum, and transmission after contact with infected animals or with drinking water contaminated by human or animal waste has been reported previously (11). In regions where *Cryptosporidium* infection is endemic, there is heterogeneity in clinical courses and outcomes. In an eight-site multicenter international study of enteric infection and malnutrition (MAL-ED), the rate of *Cryptosporidium* infection, age of onset, number of repeat infections, and clinical manifestation differed significantly by site (9). In a recent study in Dhaka, Bangladesh, we found that two-thirds of children living in an urban slum were infected with *Cryptosporidium* by 2 years of age and that one-fourth had had more than one episode of cryptosporidiosis. Fully three-fourths of the infections were subclinical, but, regardless of the symptoms, children with cryptosporidiosis were more likely to become malnourished by 2 years of age (4). Potential explanations for the *Cryptosporidium* infection heterogeneity include differences in the pathogenicity of various *Cryptosporidium* species or genotypes (12) and in host genetic susceptibility.

Candidate gene studies identified an increased risk of *Cryptosporidium* infection associated with specific alleles in HLA class I and II genes and with single nucleotide polymorphisms (SNPs) in the mannose binding lectin (*MBL*) gene (13–15). Bangladeshi preschool children with multiple *Cryptosporidium* infections (≥2 infections) were more likely to carry the -221 *MBL2* promoter variant (rs7906206; odds ratio [OR] = 4.02, *P* = 0.025) and to have the YO/XA haplotype (OR = 4.91), as well as to be deficient in their MBL serum levels (OR = 10.45) (14). Since the findings with respect to the *MBL* and HLA alleles explained *Cryptosporidium* susceptibility only partially, we conducted a genome-wide association study (GWAS) of cryptosporidiosis occurring in the first year of life using three existing birth cohorts of children in Dhaka, Bangladesh: the Performance of Rotavirus and Oral Polio Vaccines in Developing Countries (PROVIDE) study, the Dhaka Birth Cohort (DBC), and the Cryptosporidiosis Birth Cohort (CBC).

(This article was submitted to an online preprint archive [16].)

## RESULTS

Across these three cohorts, there were a total of 183 children with at least one symptomatic (diarrheal) sample that tested positive for *Cryptosporidium* within the first year of life (“cases”) (Table 1). A total of 873 children did not test positive for *Cryptosporidium* in either symptomatic (diarrheal) or surveillance samples within the first year of life (“controls”). There were no significant differences in length-for-age Z-score (LAZ) at birth (LAZ_birth_), the number of days exclusively breastfed, or sex between cases and controls (*P* > 0.05). To control for a possible role of malnutrition affecting susceptibility to infection, we compared the LAZ at 12 months of age (LAZ_12_) between cases and controls. We observed increased levels of stunting in cases (lower LAZ_12_) versus controls within PROVIDE (*P = *0.007) and CBC (*P = *0.02), while no differences were observed in stunting between cases and controls in DBC (*P = *0.97). Additionally, there was no statistically significant evidence of heterogeneity in LAZ_12_, number of days exclusively breastfed, or sex between the three studies (heterogeneity *P* [*P*_het_], >0.05).

**TABLE 1:** Demographics of study populations

Parameter	Value for:									
	Dhaka Birth Cohort (DBC)			PROVIDE			Cryptosporidiosis Birth Cohort (CBC)			
	Mean for controls (*n* = 267)	Mean for cases (*n* = 46)	*P*	Mean for controls (*n* = 354)	Mean for cases (*n* = 60)	*P*	Mean for controls (*n* = 252)	Mean for cases (*n* = 77)	*P*	*P*_het_
LAZ at 12 mos	−1.75	−1.74	0.97	−1.40	−1.79	7.28 × 10^−3^	−1.34	−1.63	0.02	0.12
Exclusive breast feeding (no. of days)	130.2	114.6	0.16	127.2	112.1	0.06	110.9	103.7	0.42	0.74
Sex (% female subjects)	46.3	34.8	0.15	45.9	46.7	0.91	52.8	57.7	0.45	0.28

### GWAS of cryptosporidiosis within the first year of life.

**FIG 1:**
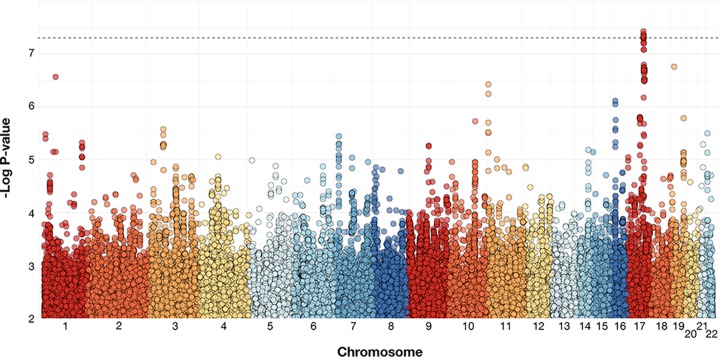
Manhattan plot for cryptosporidiosis within the first year of life. Each dot indicates the association of a single SNP with cryptosporidiosis in the first year of life. SNPs are sorted by chromosome (each color) and position along the *x* axis. The *y* axis is the -log10 *P* value for the SNP association in the meta-analysis of study-specific logistic regressions adjusting for length-for-age Z-score at 12 months, the first two study-specific principal components, and the genotyping batch for the Dhaka Birth Cohort (DBC). Genome-wide significance (5 × 10^−8^) is denoted by the dashed line. This plot is limited to associations with a *P* value below 0.01.

**FIG 2:**
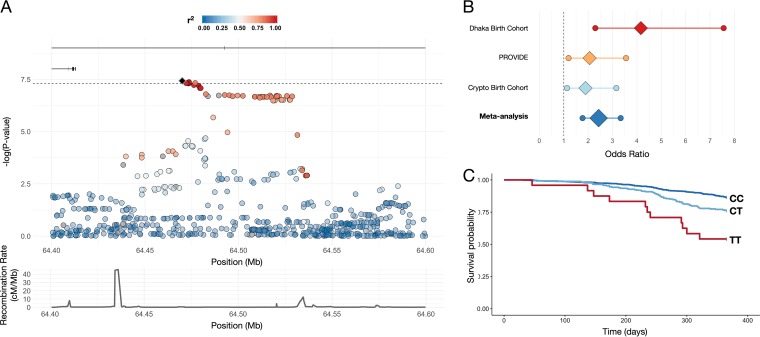
Association between variants in PRKCA and cryptosporidiosis. (A) Regional association on chromosome 17 between variants in *PRKCA* and cryptosporidiosis. Fill denotes linkage disequilibrium (*r*^2^) between the top SNP (rs58296998) and surrounding SNPs. cM/Mb, centimorgan/megabase. (B) Forest plot of odds ratios and 95% confidence intervals for top signal rs58296998 by individual cohort and meta-analysis. Crypto Birth Cohort, Cryptosporidiosis Birth Cohort. (C) Survival analysis of first episode of cryptosporidium-associated diarrhea among all participants by rs58296998 genotype within the first year of life.

We tested the association between 6.5 million SNPs across the human genome and symptomatic *Cryptosporidium* infection in the first year of life. Effects were estimated separately for the three birth cohorts and subsequently combined using a fixed-effects meta-analysis, filtered for heterogeneity (*P*_het_), minor allele frequency (MAF) (>5%), and imputation quality (INFO; score, >0.6) (Fig. 1; see also Fig. S1 in the supplemental material). A total of 6 SNPs in an intron of *PRKCA* (protein kinase c, alpha) were significantly associated with *Cryptosporidium* infection (*P* < 5 × 10^−8^) (Fig. 2A). For the SNP most highly associated with *Cryptosporidium* infection (rs58296998), each copy of the risk allele (T) conferred 2.4 times the odds of cryptosporidiosis within the first year of life (*P* = 3.73 × 10^−8^). The effect size and risk allele were consistent across all three studies (*P*_het_ value of 0.11) (Fig. 2B). After conditioning performed on the basis of rs58296998 (by including this SNP in the logistic regression model as a covariate), the evidence for association with the remaining SNPs in the region was no longer statistically significant, suggesting that the observed association in *PRKCA* is explained by a single SNP (rs58296998) or by one highly correlated with this SNP (Fig. S2A). Among the 26 children homozygous for the risk allele (TT) at rs58296998, 46% developed symptomatic cryptosporidiosis during the first year of life. This proportion decreased to 24% for children heterozygous (CT) for this risk allele (*n* = 272), compared to 13% of children homozygous (CC) for the risk allele (*n* = 745).

10.1128/mBio.03343-19.1 FIG S1 Quality control workflow for all three cohorts. Download FIG S1, PDF file, 0.1 MB. Copyright © 2020 Wojcik et al. 2020 Wojcik et al. This content is distributed under the terms of the Creative Commons Attribution 4.0 International license.

10.1128/mBio.03343-19.2 FIG S2 Characteristics of *PRKCA* region and top SNP. (A) Regional association in *PRKCA* region after conditioning with top signal rs58296998, showing significantly diminishment between recombination peaks. (B) Survival analysis of the first episode of cryptosporidiosis associated with the PRKCA rs58296998 genotype within the first year of life among cases. Adjusting for the study, we saw no additive relationship between an additive model of the risk allele (T) with genotypes having no, one, or two copies of the T allele and earlier infection (*P* = 0.095). (C) Relationship between genotype for PRKCA SNP rs58296998 and severity of diarrhea as determined by Ruuska score within PROVIDE. Under an additive model, we saw a statistically significant relationship between PRKCA genotypes and diarrhea severity (*P* = 0.028). Download FIG S2, PDF file, 2.8 MB. Copyright © 2020 Wojcik et al. 2020 Wojcik et al. This content is distributed under the terms of the Creative Commons Attribution 4.0 International license.

**FIG 3:**
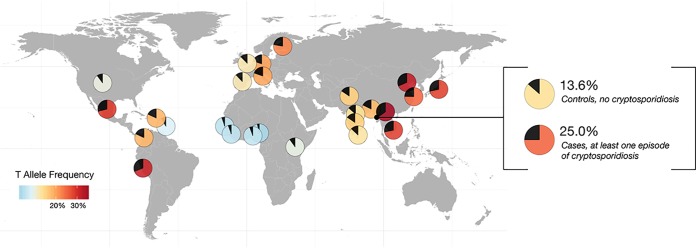
Allele frequencies for allele T at top signal rs58296998 as determined by analysis of 1000 Genomes phase 3 data, as well as by analysis of case/control status in the three cohorts combined. Each pie chart on the map shows the frequency of the T allele with the black wedge. The remainder of each pie chart is colored in accordance with that T allele frequency. The inset provides the T allele frequency for children without any symptomatic cryptosporidiosis in the first year of life (controls; MAF = 13.6%) and for those with at least one diarrheal episode (cases; MAF = 25.0%).

The rs58296998 T allele frequencies (15.0% to 16.7%) for all three cohorts in this region are consistent with the Bangladeshi reference population (1000 Genomes phase 3) frequency of 18% and the overall South Asian frequency of 15% (17). Globally, the highest frequencies of rs58296998 T allele are found in East Asian populations, with the highest T allele frequency of 34% of the Chinese Dai in Xishuangbanna, China. The rs58296998 T allele is at lower frequencies within Africa, at 9% within the Luhya in Kenya, and is even less frequent in West Africa (3.5% to 5.5%) (Fig. 3).

Cases had their first diarrheal episode positive for *Cryptosporidia* at a mean of 242 days of age. We confirmed the GWAS results with respect to the dosage of rs58296998 risk alleles significantly associated with time to first diarrheal sample positive for *Cryptosporidia* among cases versus right-censored controls (up to the child’s first birthday) (*P* = 6.37 × 10^−8^). All children homozygous for the risk allele (TT) had their first episode in the first year of life (Fig. 2C). Among cases, however, there was no statistically significant association between rs58296998 genotype and time to infection (*P* = 0.095) (Fig. S2B). In PROVIDE, the rs58296998 genotype was associated with severity of diarrhea as determined by the Ruuska score (*P = *0.028) (Fig. S2C).

Suggestive SNP associations with *Cryptosporidium* (*P < *10^−6^) were also identified on chromosome 11 (chr11) and chr16. The strongest association on chromosome 11 (rs4758351) was found within an intergenic region of a cluster of olfactory receptor genes. Each copy of the rs4758351 A allele (MAF of 14%) conferred 2.39 times the odds of *Cryptosporidium* within the first year of life (*P* = 3.78 × 10^−7^) (Fig. S3A). Multiple SNPs in this region of chr11 (position 6015194 to position 6024551) had similar magnitudes and strengths of association with *Cryptosporidium* (OR, 2.13 to 2.39). The strongest association on chromosome 16 was with the rs9937140 SNP, located upstream of apolipoprotein O pseudogene 5 (*APOOP5*). Each copy of the rs9937140 G allele (MAF, 23%) conferred 1.99 times the odds of cryptosporidiosis (*P* = 7.75 × 10^−7^) (Fig. S3B).

10.1128/mBio.03343-19.3 FIG S3 LocusZoom plots of suggestive signals for GWAS. (A) LocusZoom plot of suggestive signal on chromosome 11 (rs4758351). Each dot represents a single SNP association from the meta-analysis of the three study-specific logistic regressions adjusting for HAZ (11), the first two study-specific principal components, and the batch for the DBC. The *x* axis represents the physical position along chromosome 11 with the gene locations indicated below. The *y* axis represents the log *P* value from the single SNP association. The fill represents the level of linkage disequilibrium (*r*^2^) between the top signal (rs4758351) and the surrounding SNPs. (B) LocusZoom of suggestive signal on chromosome 16 (rs9937140). Each dot represents a single SNP association from the meta-analysis of the three study-specific logistic regressions adjusting for HAZ (11), the first two study-specific principal components, and the batch for DBC. The *x* axis represents the physical position along chromosome 16 with the gene locations below. The *y* axis represents the log *P* value from the single SNP association. The fill represents the level of linkage disequilibrium (*r*^2^) between the top signal (rs9937140) and surrounding SNPs. Download FIG S3, PDF file, 0.1 MB. Copyright © 2020 Wojcik et al. 2020 Wojcik et al. This content is distributed under the terms of the Creative Commons Attribution 4.0 International license.

### Expression and PrediXcan.

We used a publicly available resource, the Genotype-Tissue Expression (GTEx) Project, to estimate the influence of human genetic variation on human gene expression in multiple tissues (18, 19). The associated rs58296998 SNP, located in the *PRKCA* gene, is also associated with *PRKCA* expression. This expression quantitative trait locus (eQTL), or a genetic variant previously shown to influence the expression of a gene, showed decreasing expression of *PRKCA* with each T allele in the esophageal muscularis (*P *= 3.12 × 10^−5^), the sigmoid colon (*P = *4.61 × 10^−4^), and the esophageal mucosa (*P = *7.50 × 10^−4^) (19). These expression data, coupled with the GWAS result, suggested that decreased expression of *PRKCA* is correlated with increased risk of symptomatic *Cryptosporidium* infection within the first year of life.

### Additional genome-wide expression and gene set analyses.

**FIG 4:**
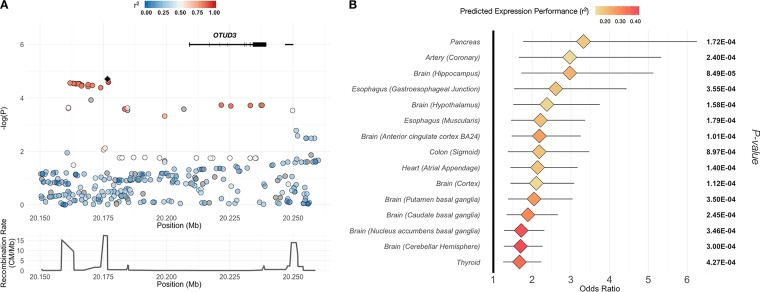
OTUD3 region showing association with cryptosporidiosis in the first year of life. (A) Association of SNPs on chromosome 1 region, colored by linkage disequilibrium (*r*^2^) with index SNP (black diamond). (B) Association of case status with imputed gene expression in all tissues with *P* value of <0.001 and predicted expression performance of *r*^2^ = >0.1.

In the absence of direct gene expression measurement, we relied on previously estimated tissue-specific associations between genome-wide SNPs and gene expression, which quantify the genetic component of gene expression. We estimated predicted patterns of genome-wide differential gene expression between cases and controls by weighting the summary statistics from our GWAS of cryptosporidiosis in the first year of life by the use of tissue-specific PredictDB weights. These SNP-level estimates were then combined for each gene to infer association between imputed gene expression and cryptosporidiosis (20, 21). No association of predicted gene expression with cryptosporidiosis reached statistical significance. A total of 13 genes showed a nominally significant (*P* < 0.001) association in more than one tissue-specific model (see Table S1 in the supplemental material; see also Fig. S4). Variants in the gene *OTUD3* (OTU deubiquitinase 3) (chr1; position 20208356 to position 20239438) were associated with cryptosporidiosis in 18 different tissue-specific models (*P* < 0.001). In all tissue-specific models, individuals with predicted increased expression of *OTUD3* had an increased risk of cryptosporidiosis within the first year of life (OR, 1.68 to 6.63; *P* = 8.46 × 10^−5^ to 8.97 × 10^−4^) (Fig. 4).

10.1128/mBio.03343-19.4 FIG S4 Shared associations for predicted gene expression, filtered for gene-tissue pairs with *P* values of <0.001. Download FIG S4, PDF file, 0.2 MB. Copyright © 2020 Wojcik et al. 2020 Wojcik et al. This content is distributed under the terms of the Creative Commons Attribution 4.0 International license.

10.1128/mBio.03343-19.8 TABLE S1 Results for metaXcan analysis evaluating association of predicted gene expression with cryptosporidiosis in the first year of life. Download Table S1, XLSX file, 0.03 MB. Copyright © 2020 Wojcik et al. 2020 Wojcik et al. This content is distributed under the terms of the Creative Commons Attribution 4.0 International license.

We also performed gene set enrichment analysis using MSigDB hallmark gene sets (*n* = 50), KEGG (*n* = 186) and BioCarta (*n* = 217) by combining gene-level summary statistics to examine aggregate signals within biological pathways. No pathways reached statistical significance after adjusting for multiple comparisons; however, data from several gene sets were suggestive (Table S2). The two top-ranked gene sets are among the hedgehog signaling pathways, namely, the hallmark hedgehog signaling pathway (empirical *P* value [*P*_emp_] = 5.04 × 10^4^) (Bayes factor [BF] = 515.65) and KEGG hedgehog signaling pathway (*P*_emp_ = 1.47 × 10^−3^) (BF = 235.59).

10.1128/mBio.03343-19.9 TABLE S2 Data from gene-set analysis determined on the basis of metaXcan results for association of predicted gene expression with cryptosporidiosis in the first year of life. Download Table S2, XLSX file, 0.03 MB. Copyright © 2020 Wojcik et al. 2020 Wojcik et al. This content is distributed under the terms of the Creative Commons Attribution 4.0 International license.

## DISCUSSION

Here, we present the results of the first genome-wide association study of symptomatic *Cryptosporidium* infection. Specifically, we tested the role of host genetics in susceptibility to *Cryptosporidium* infection associated with diarrhea within the first year of life. A region on chromosome 17 was identified, with each additional T allele of rs58296998, an intronic SNP in *PRKCA*, conferring 2.4 times the odds of cryptosporidiosis within the first year of life. Additionally, this SNP was previously identified as an eQTL of *PRKCA*, with decreased expression of *PRKCA* associated with the T allele. This suggests that this SNP may influence *Cryptosporidium* infection through decreased expression of *PRKCA*.

The protein kinase C alpha gene (*PRKCA*) is an isotype of the protein kinase C (PKC) family, whose members are serine and threonine specific and are known to be involved in diverse cellular signaling pathways. Specifically, PKCs have numerous roles in the development and function of the gastrointestinal tract (22) and in the immune response (23). This relationship was confirmed with knockout experiments, where PKCα was shown to be a positive regulator of Th17 cell effector functions. PKCα-deficient [Prkca(−/−)] cells failed to produce the appropriate levels of interleukin-17A (IL-17A) *in vitro* (23). An analysis of Cryptosporidium parvum*-*infected mice demonstrated the importance of the Th17 response to infection, showing increased levels of IL-17 mRNA and Th17 cell-related cytokines in gut tissue after infection (24). Additionally, both pharmacological inhibition and genetic PKCα inhibition have been shown to prevent NHE3 internalization, Na^+^ malabsorption, and tumor necrosis factor (TNF)-mediated diarrhea, despite continued barrier dysfunction (25), supporting the idea of a role for *PRKCA* in symptomatic cryptosporidiosis. This link between *PRKCA* and Th17 may be critical to gut infections and, specifically, to infection of *Cryptosporidium* in the developing infant gut. We identified a SNP that was associated with decreased expression of *PRKCA* and thus was less able to mediate the IL-17 immune response during *Cryptosporidium* infection. *PRKCA* has also been shown to be associated with numerous other infections, including infections by Staphylococcus aureus (26); with progression of sepsis (27) and toxoplasmosis (28); with Burkholderia cenocepacia infections in cystic fibrosis patients (29); and with hepatitis E virus replication (30).

As an obligate intracellular parasite, *Cryptosporidium* relies on host cells to complete its life cycle in the human host; thus, it is also plausible that *PRKCA* directly mediates susceptibility via impacts on parasite invasion. Sporozoites invade brush border intestinal epithelial cells by inducing volume increases (31) and cytoskeletal remodeling at the site of host cell attachment (32), leading to engulfment via host membrane protrusions. Studies have shown that inhibition of host factors, including actin remodeling proteins and PKC enzymes, is sufficient to inhibit sporozoite invasion *in vitro* (32). Interestingly, PKCα has been shown to play an important role in Escherichia coli pathogenesis (33). Like *Cryptosporidium*, E. coli induces host actin condensation at the site of host cell invasion, and immunocytochemical studies indicate that activated PKCα colocalized with actin condensation at the bacterial entry site (34).

While our top SNP within *PRKCA* has previously been shown to influence the expression of *PRKCA* in GTEx, our imputed gene expression analysis using PrediXcan did not reveal a significant difference in predicted levels of *PRKCA* expression between cases and controls. This was likely due to the difference between a single SNP being examined in GTEx and the combined effects of multiple eQTLs estimated from a European descent reference population in PrediXcan. A major limitation of predicted gene expression analyses is the lack of population specificity for non-European groups (35). The PrediXcan models were derived from individuals of European descent, as were the covariance structures used to infer correlations between eQTLs. We saw a direct relationship between population differences in allele frequencies for the weighted SNPs and impaired performance. Specifically, we observed the lowest predictive performance in tissues for which the informative SNPs had large differences in allele frequencies between European and South Asian populations in the 1000 Genomes Project phase 3 data (17) (see Fig. S5 in the supplemental material). These included two tissues, namely, esophageal mucosa and the colon sigmoid tissue, in which rs58296998 was identified as an eQTL for *PRKCA*. These trends highlight the importance of reference populations representative of global populations to ensure that tools are useful in non-European populations, such as ours. We also identified an association of increased expression of *OTUD3* with increased odds of cryptosporidiosis within the first year of life. This gene is associated with ulcerative colitis (36–42) and inflammatory bowel disease (43, 44). This finding is consistent with the hypothesis of a pathway shared between enteric infection and autoimmune intestinal disease, as indicated in a previous genetic analysis of Entamoeba histolytica infection in the same study population (45).

10.1128/mBio.03343-19.5 FIG S5 Gene expression prediction characteristics of *PRKCA*. (A) Correlation per tissue of differences in allele frequencies between European and South Asian populations with prediXcan weights for PRKCA. We saw that there is a statistically significant correlation (*P* < 0.05) in the tissues of interest: colon sigmoid and esophagus mucosa. (B) Correlation per tissue between weights and frequency differences versus the predictive performance in our participants. The tissues of interest (colon sigmoid, esophagus mucosa) show high correlation and low predictive performance *r*^2^. Fill indicates the log *P* value for correlation. (C) Difference per SNP between European and South Asian allele frequencies (EUR-SAS) versus the prediXcan weight. Fill indicates the South Asian allele frequencies. We note that many of the highest-weighted alleles are of low frequency or absent in South Asia. Download FIG S5, PDF file, 0.1 MB. Copyright © 2020 Wojcik et al. 2020 Wojcik et al. This content is distributed under the terms of the Creative Commons Attribution 4.0 International license.

Collapsing the predicted patterns of differentially expressed genes into gene sets, we found enrichment in the hedgehog signaling pathway. A previous study examined the gene expression profiles of long noncoding RNA (lncRNA) and mRNA in HCT-8 cells infected with C. parvum subtype IId (46). Of note, *PRKCA* was the most significantly differentially expressed gene in infected HCT8 cells 24 h postinfection (2.24-fold decreased expression in infected cells; *P* = 3.82 × 10^−5^). Pathway analysis of the differentially expressed mRNAs found that genes in the hedgehog signaling pathway were significantly enriched during *Cryptosporidium* infection. This finding, in combination with our identification of hedgehog signaling in imputed gene expression profiles, is suggestive of a potential link between decreased *PRKCA* expression and hedgehog signaling; however, further research to confirm these findings and to elucidate the role of *PRKCA* genetic variation in gene expression and hedgehog pathway perturbation is needed.

A potential limitation of our study was that, due to the use of sensitive molecular diagnostics, multiple enteropathogens were frequently detected in each diarrheal sample. However, we did not detect the same genetic signatures as that seen in our previous study of Entamoeba histolytica in this same study population for *Cryptosporidium* (45). Further, coinfection with multiple pathogens would dilute the statistical signal for any one pathogen, and yet we found a statistically significant result for *Cryptosporidium*. Therefore, we are confident that our results are specific to cryptosporidiosis, despite cooccurrence with other enteric pathogens.

Through a GWAS meta-analysis of three separate birth cohorts, we identified a region in *PRKCA* on chromosome 17 as being associated with increased risk of symptomatic cryptosporidiosis in the first year of life among Bangladeshi infants. This gene has previously been implicated in other infectious outcomes, indicating pleiotropy with the immune system’s reaction to numerous pathogens. Publicly available data support a link between our top SNP and expression of *PRKCA*, suggesting a mechanism operating via Th17 inflammatory control. Clinical trials are currently proposed for PKC isotypes, including PKC-alpha, for treatment of autoimmune disease (47). These treatments may also be important for cryptosporidiosis, which lacks treatment for young children, due to an underlying shared pathway identified in this study. Identifying host genetic variations associated with cryptosporidiosis, such as those in *PRKCA*, can help us identify viable drug targets to improve treatment and prevention of this major cause of morbidity and mortality. Further research is needed to elucidate the mechanism underlying this relationship and to better understand the complex interplay of genetic susceptibility and environmental influences in the development of intestinal disease.

## MATERIALS AND METHODS

### Study protocol.

The study protocol was approved by the Research and Ethical Review Committee of the International Center for Diarrheal Disease Research, Bangladesh, and by the Institutional Review Board of the University of Virginia and the Institutional Review Board of the Johns Hopkins Bloomberg School of Public Health. The parents or guardians of all individuals provided informed consent.

### Dhaka Birth Cohort study design.

Designed to study the influence of malnutrition in child development, the Dhaka Birth Cohort (DBC) is a subset of a larger birth cohort recruited from the urban slum in the Mirpur Thana in Dhaka, Bangladesh. Children were enrolled within the first week after birth and followed up biweekly with household visits by trained field research assistants (FRAs) for the first year of life. Anthropometric measurements were collected at the time of enrollment and every 3 months thereafter. Length-for-age adjusted Z-scores (LAZ) were calculated by comparing the lengths and weights of study subjects with those of the World Health Organization (WHO) reference population, adjusting for age and sex, using WHO Anthro software, version 3.0.1. Field research assistants (FRAs) collected diarrheal stool samples from the home or study field clinic every time that the mother of the child reported diarrhea. To maintain a cold chain, the samples were transported to the Centre for Diarrheal Disease Research, Bangladesh (ICDDR,B) parasitology laboratory. The presence of *Cryptosporidium* was determined using enzyme-linked immunosorbent assay (ELISA). More details can be found in previously published reports by Steiner et al. (4) and Korpe et al. (9). We used a nested case-control design, where children with at least one diarrheal sample positive for *Cryptosporidium* within the first year were defined as “cases.” Children with diarrheal samples that were not positive for *Cryptosporidium* were defined as “controls.”

### PROVIDE study design.

The “Performance of Rotavirus and Oral Polio Vaccines in Developing Countries” (PROVIDE) Study consists of a randomized controlled clinical trial and birth cohort from the same urban slum in the Mirpur Thana in Dhaka, Bangladesh, as the DBC and Cryprosporidiosis Birth Cohort (CBC) (see below). PROVIDE was specifically designed to assess the influence of various factors on oral vaccine efficacy among children in areas with high poverty, urban overcrowding, and poor sanitation. The 2-by-2 factorial design looked specifically at the efficacy of the 2-dose Rotarix oral rotavirus vaccine and oral polio vaccine (OPV) with an inactivated polio vaccine (IPV) boost over the first 2 years of life. All participants were from the Mirpur area of Dhaka, Bangladesh, with pregnant mothers recruited from the community by female Bangladeshi FRAs. Each participant had 15 scheduled follow-up clinic visits, as well as biweekly diarrhea surveillance through home visits by FRAs. The presence of *Cryptosporidium* in diarrheal samples was determined by ELISA. Consistently with the DBC phenotype definition, cases had at least one diarrheal sample positive for *Cryptosporidium* within the first year of life. Controls had at least one diarrheal sample available for testing, but none were positive for *Cryptosporidium*. Severity of diarrhea was determined with the Ruuska score, which assesses severity as a function of diarrhea length, clinical symptoms, and other clinical features (48).

### Cryptosporidiosis Birth Cohort study design.

The Cryptosporidiosis Birth Cohort (“Cryptosporidiosis and Enteropathogens in Bangladesh”; ClinicalTrials.gov registration no. NCT02764918) is a prospective longitudinal birth cohort study in two sites in Bangladesh. The first site is in an urban, economically depressed neighborhood of Mirpur, and the second is in Mirzapur, a rural subdistrict 60 km northwest of Dhaka. The two birth cohorts were established in parallel, with the objective of understanding the incidence of cryptosporidiosis, the acquired immune response, and host genetic susceptibility to cryptosporidiosis in Bangladeshi children. Pregnant women were recruited and screened, and infants were enrolled at birth. Participants were followed twice-weekly with in-home visits to monitor for child morbidity and diarrhea for 24 months. Infant length and weight were measured every 3 months, and weight-for-age and length-for-age adjusted Z-scores were determined using World Health Organization Anthro software (version 3.2.2). Stool samples were collected during diarrheal illness and once per month for surveillance. Stool was tested for *Cryptosporidium* by quantitative PCR (qPCR) assay modified from a method reported previously by Liu et al. (49). A cycle threshold value of 40 was used. The pan-*Cryptosporidium* primers and probes target the 18S gene in multiple species known to infect humans (4).

### Genotype data.

DNA for all three cohorts was extracted from blood samples collected in the first few months of follow-up. The Dhaka Birth Cohort (DBC) and PROVIDE Study data were generated and cleaned as described previously (45). A summary of quality control (QC) procedures is provided in Fig. S1 in the supplemental material. Briefly, a total of 396 children in the DBC were genotyped on three different Illumina arrays. Imputation to 1000Genomes phase 3 data was performed for all individuals. After postimputation QC, which included additional filtering for relatedness and for poorly imputed variants, a total of 396 individuals and 10.2 million SNPs were included in the DBC data freeze. For PROVIDE, a total of 541 individuals were genotyped on a Multi-Ethnic Genotyping Array (MEGA) (Illumina). After standard quality control measures (including the use of minor allele frequency values of >0.5% and missingness values of <5%) were applied and first-degree-related individuals removed, a total of 499 individuals remained. After imputation to 1000Genomes and subsequent postimputation QC, a total of 499 individuals and 10.8 million genetic variants remained. For CBC, a total of 630 individuals were genotyped on a Multi-Ethnic Global Array (MEGA) (Illumina). One individual was removed for first-degree relatedness (PI_HAT > 0.2), 31 individuals were removed as PCA outliers, and 3 individuals were removed for heterozygosity. No individuals or SNPs were removed for missingness (>5%). Additional SNP-level filters included the use of minor allele frequency (MAF) values of <0.5% (M = 751,869) and Hardy-Weinberg equilibrium *P* values of <10^−5^ (M = 85). After all QC steps, CryptoCohort genotype data included 594 individuals and 826,228 SNPs. Phasing in of SHAPEIT2 (50) was followed by imputation to 1000 Genomes phase 3 data (1000Genomes) (17) performed with IMPUTE2 (51, 52). All three studies were separately imputed to 1000Genomes.

### Cross-study genetic data harmonization.

After imputation, all three data sets (DBC, PROVIDE, and CBC) were double-checked for relatedness (both within each study and between studies) to ensure independence. One individual from each pair of related individuals was dropped in a manner consistent with the first or second degree of relatedness (PI_HAT > 0.2). Individual outliers for heterozygosity (*F* = >5 standard deviations from the mean) were also excluded from further analysis. A total of 85 individuals were dropped from DBC, 9 from PROVIDE, and 34 from CBC. Only the top principal component from the combined data set was found to be significantly associated with outcome (Fig. S6).

10.1128/mBio.03343-19.6 FIG S6 Quality control metrics for combined GWAS. (A) Distribution of three studies for principal components 1 to 5, colored by study. Crypto (red), Cryptosporidiosis Birth Cohort (CBC); DBC (green), Dhaka Birth Cohort; provide (blue), PROVIDE Study. (B) Distribution of three studies for principal components 1 to 5, colored by case status. Cases are shown in blue and controls in red. Only the first principal component was significantly associated with case status. (C) Histogram of heterozygosity distribution by cohort. Here, we show the distribution of heterozygosity by cohort. crypto, Cryptosporidiosis Birth Cohort; dbc, Dhaka Birth Cohort; provide, PROVIDE Study. The data on the *x* axis represent *F*, or the coefficient of heterozygosity. Download FIG S6, PDF file, 0.2 MB. Copyright © 2020 Wojcik et al. 2020 Wojcik et al. This content is distributed under the terms of the Creative Commons Attribution 4.0 International license.

### Statistical analysis.

All three studies (DBC, PROVIDE, and CBC) were analyzed separately using logistic regression with an additive model accounting for imputed genotype weights in SNPTEST (51, 53, 54). All three analyses were adjusted for length-for-age Z-score (LAZ) at 1 year of age, for sex, and for the first two principal components. The Dhaka Birth Cohort was additionally conditioned on the genotyping array to account for batch effects. We combined the three analyses in a fixed-effects meta-analysis within META. Results were filtered for *P*_het_ values of >0.05, minor allele frequency (MAF) of >5%, and INFO score of >0.6 in all three studies, resulting in 6,504,706 SNPs. The conditional analyses were run separately by cohort for the *PRKCA* region, with each analysis being conditioned on rs58296998 in addition to the original covariates with SNPTEST. Results were again filtered for heterogeneity or *P*_het_ values of >0.05, MAF of >5%, and INFO score of >0.6 in all three studies.

### Allele frequencies.

The allele frequencies were derived from the 1000 Genomes Project phase 3 data, v5a (17). Individuals were stratified by their denoted population with first degree related individuals removed.

### GTEx and eQTL overlap GWAS results.

Expression quantitative trait loci (eQTLs) were identified through the use of the GTEx Portal (https://www.gtexportal.org/home/) on 6 August 2018 (19). The top SNP was identified as an eQTL for *PRKCA* with *P* values of <0.001 for multiple tissues. PrediXcan measured gene expression in 48 tissues and subsequently mapped genetic variation across the human genome to tissue-specific gene expression levels. Therefore, eQTLs are identified in a tissue-specific manner and annotated as such on the GTEx Portal.

### MetaXcan imputation and association analysis.

To impute gene expression and association with outcome from our GWAS summary statistics, we applied MetaXcan (S-PrediXcan and packaged best practices) (21). Weights were previously derived with GTEx v7 data in a population of subjects of European descent, with accompanying European-descent linkage disequilibrium metrics for the SNP covariance matrices (PredictDB Data Respository; http://predictdb.org/). MetaXcan was used instead of the original PrediXcan to ensure consistency in models with our GWAS. All 48 tissues were run separately for the meta-analysis results previously described. Following imputation and estimation of gene expression with outcome, we calculated weights for each gene-tissue pair as the ratio between the number of SNPs used in the model and the total number that were prespecified in the model multiplied by predicted expression performance. To determine associations across many tissues, a *P* value threshold of 0.001 was utilized. A strict Bonferroni correction performed for the 242,686 comparisons resulted in a *P* value threshold of 0.05/242,686 = 2.06 × 10^−7^, according to which no comparison yielded a statistically significant result. The relationships of allele frequencies in European and South Asian populations with PrediXcan weights were examined to assess prediction capacity (Fig. S5 and S7).

### Gene set enrichment analysis.

Gene set enrichment analysis was conducted on the described previously imputed gene expression data summary statistics from MetaXcan. For each gene, we selected the tissue corresponding to the smallest *P* value. Using the program GIGSEA (Genotype Imputed Gene Set Enrichment Analysis [55]), we tested for associations of 453 curated gene sets defined by MSigDB hallmark gene sets (56), as well as KEGG (Kyoto Encyclopedia of Genes and Genomes; https://www.kegg.jp) and BioCarta (57) gene sets (58). To account for redundancy with overlapping gene sets, we utilized the weighted multiple linear regression model, using the matrix operation to increase speed, with a total of 1,000 permutations. A false-discovery rate of 0.05 was calculated on the ranked results.

### Data availability.

Data are publicly available from the NIH, via dbGAP, phs001478.v1.p1 (Exploration of the Biologic Basis for Underperformance of Oral Polio and Rotavirus Vaccines in Bangladesh), or by request from us. All analysis programs used are detailed above, but the actual code in R for each analysis is also available by request from us.

10.1128/mBio.03343-19.7 FIG S7 Gene expression prediction characteristics of OTUD3. Data represent correlation per tissue of differences in allele frequencies between European and South Asian populations with prediXcan weights for OTUD3 and correlation per tissue between weights and frequency differences versus the predictive performance in our participants. The tissues of interest (colon sigmoid, esophagus mucosa) show high correlation and low predictive performance (*r*^2^). Fill indicates the log *P* value for correlation. Data represent difference per SNP between European and South Asian allele frequencies (EUR-SAS) versus the prediXcan weight. Fill indicates the South Asian allele frequencies. We note that many of the highest-weighted alleles are of low frequency or absent in South Asia. Download FIG S7, PDF file, 0.1 MB. Copyright © 2020 Wojcik et al. 2020 Wojcik et al. This content is distributed under the terms of the Creative Commons Attribution 4.0 International license.

## References

[1] Sow SO, Muhsen K, Nasrin D, Blackwelder WC, Wu Y, Farag TH, Panchalingam S, Sur D, Zaidi AKM, Faruque ASG, Saha D, Adegbola R, Alonso PL, Breiman RF, Bassat Q, Tamboura B, Sanogo D, Onwuchekwa U, Manna B, Ramamurthy T, Kanungo S, Ahmed S, Qureshi S, Quadri F, Hossain A, Das SK, Antonio M, Hossain MJ, Mandomando I, Nhampossa T, Acácio S, Omore R, Oundo JO, Ochieng JB, Mintz ED, O'Reilly CE, Berkeley LY, Livio S, Tennant SM, Sommerfelt H, Nataro JP, Ziv-Baran T, Robins-Browne RM, Mishcherkin V, Zhang J, Liu J, Houpt ER, Kotloff KL, Levine MM 2016 The burden of cryptosporidium diarrheal disease among children <24 months of age in moderate/high mortality regions of sub-Saharan Africa and South Asia. PLoS Negl Trop Dis 10:e0004729. doi:10.1371/journal.pntd.0004729. 27219054 PMC4878811

[2] Guerrant DI, Moore SR, Lima AA, Patrick PD, Schorling JB, Guerrant RL 1999 Association of early childhood diarrhea and cryptosporidiosis with impaired physical fitness and cognitive function four–seven years later in a poor urban community in northeast Brazil. Am J Trop Med Hyg 61:707–713. doi:10.4269/ajtmh.1999.61.707. 10586898

[3] Mondal D, Haque R, Sack RB, Kirkpatrick BD, Petri WA 2009 Attribution of malnutrition to cause-specific diarrheal illness: evidence from a prospective study of preschool children in Mirpur, Dhaka, Bangladesh. Am J Trop Med Hyg 80:824–826. doi:10.4269/ajtmh.2009.80.824. 19407131 PMC3410540

[4] Steiner KL, Ahmed S, Gilchrist CA, Burkey C, Cook H, Ma JZ, Korpe PS, Ahmed E, Alam M, Kabir M, Tofail F, Ahmed T, Haque R, Petri WA, Faruque A 2018 Species of cryptosporidia causing subclinical infection associated with growth faltering in rural and urban Bangladesh: a birth cohort study. Clin Infect Dis 67:1347–1355. doi:10.1093/cid/ciy310. 29897482 PMC6186860

[5] Korpe PS, Haque R, Gilchrist C, Valencia C, Niu F, Lu M, Ma JZ, Petri SE, Reichman D, Kabir M, Duggal P, Petri WA 2016 Natural history of cryptosporidiosis in a longitudinal study of slum-dwelling Bangladeshi children: association with severe malnutrition. PLoS Negl Trop Dis 10:e0004564. doi:10.1371/journal.pntd.0004564. 27144404 PMC4856361

[6] Chalmers RM, Robinson G, Elwin K, Elson R 2019 Analysis of the Cryptosporidium spp. and gp60 subtypes linked to human outbreaks of cryptosporidiosis in England and Wales, 2009 to 2017. Parasit Vectors 12:95. doi:10.1186/s13071-019-3354-6. 30867023 PMC6417012

[7] Khalil IA, Troeger C, Rao PC, Blacker BF, Brown A, Brewer TG, Colombara DV, De Hostos EL, Engmann C, Guerrant RL, Haque R, Houpt ER, Kang G, Korpe PS, Kotloff KL, Lima AAM, Petri WA, Platts-Mills JA, Shoultz DA, Forouzanfar MH, Hay SI, Reiner RC, Mokdad AH 2018 Morbidity, mortality, and long-term consequences associated with diarrhoea from Cryptosporidium infection in children younger than 5 years: a meta-analyses study. Lancet Glob Health 6:e758–e768. doi:10.1016/S2214-109X(18)30283-3. 29903377 PMC6005120

[8] Kattula D, Jeyavelu N, Prabhakaran AD, Premkumar PS, Velusamy V, Venugopal S, Geetha JC, Lazarus RP, Das P, Nithyanandhan K, Gunasekaran C, Muliyil J, Sarkar R, Wanke C, Ajjampur SSR, Babji S, Naumova EN, Ward HD, Kang G 2017 Natural history of cryptosporidiosis in a birth cohort in southern India. Clin Infect Dis 64:347–354. doi:10.1093/cid/ciw730. 28013266 PMC5241779

[9] Korpe PS, Valencia C, Haque R, Mahfuz M, McGrath M, Houpt E, Kosek M, McCormick BJJ, Penataro Yori P, Babji S, Kang G, Lang D, Gottlieb M, Samie A, Bessong P, Faruque ASG, Mduma E, Nshama R, Havt A, Lima IFN, Lima AAM, Bodhidatta L, Shreshtha A, Petri WA, Ahmed T, Duggal P 2018 Epidemiology and risk factors for cryptosporidiosis in children from 8 low-income sites: results from the MAL-ED study. Clin Infect Dis 67:1660–1669. doi:10.1093/cid/ciy355. 29701852 PMC6233690

[10] Sarkar R, Kattula D, Francis MR, Ajjampur SSR, Prabakaran AD, Jayavelu N, Muliyil J, Balraj V, Naumova EN, Ward HD, Kang G 2014 Risk factors for cryptosporidiosis among children in a semi urban slum in southern India: a nested case-control study. Am J Trop Med Hyg 91:1128–1137. doi:10.4269/ajtmh.14-0304. 25331810 PMC4257634

[11] Bennett JE, Dolin R, Blaser MJ 2015 Mandell, Douglas, and Bennett’s principles and practice of infectious diseases, 8th ed Elsevier/Saunders, Philadelphia, PA.

[12] Gilchrist CA, Cotton JA, Burkey C, Arju T, Gilmartin A, Lin Y, Ahmed E, Steiner K, Alam M, Ahmed S, Robinson G, Zaman SU, Kabir M, Sanders M, Chalmers RM, Ahmed T, Ma JZ, Haque R, Faruque ASG, Berriman M, Petri WA 2018 Genetic diversity of Cryptosporidium hominis in a Bangladeshi community as revealed by whole-genome sequencing. J Infect Dis 218:259–264. doi:10.1093/infdis/jiy121. 29514308 PMC6009673

[13] Kirkpatrick BD, Haque R, Duggal P, Mondal D, Larsson C, Peterson K, Akter J, Lockhart L, Khan S, Petri WA 2008 Association between Cryptosporidium infection and human leukocyte antigen class I and class II alleles. J Infect Dis 197:474–478. doi:10.1086/525284. 18248305 PMC3404124

[14] Carmolli M, Duggal P, Haque R, Lindow J, Mondal D, Petri WA, Mourningstar P, Larsson CJ, Sreenivasan M, Khan S, Kirkpatrick BD 2009 Deficient serum mannose-binding lectin levels and MBL2 polymorphisms increase the risk of single and recurrent Cryptosporidium infections in young children. J Infect Dis 200:1540–1547. doi:10.1086/606013. 19827946 PMC3400050

[15] Kelly P, Jack DL, Naeem A, Mandanda B, Pollok RC, Klein NJ, Turner MW, Farthing MJ 2000 Mannose-binding lectin is a component of innate mucosal defense against Cryptosporidium parvum in AIDS. Gastroenterology 119:1236–1242. doi:10.1053/gast.2000.19573. 11054381

[16] Wojcik GL, Korpe P, Marie C, Mychaleckyj J, Kirkpatrick BD, Rich SS, Concannon P, Faruque ASG, Haque R, Petri WA Jr, Duggal P 2019 Genome-wide association study of cryptosporidiosis in infants implicates PRKCA. bioRxiv doi:10.1101/819052. PMC7002356 32019797

[17] 1000 Genomes Project Consortium, Auton A, Brooks LD, Durbin RM, Garrison EP, Kang HM, Korbel JO, Marchini JL, McCarthy S, McVean GA, Abecasis GR 2015 A global reference for human genetic variation. Nature 526:68–74. doi:10.1038/nature15393. 26432245 PMC4750478

[18] eGTEx Project. 2017 Enhancing GTEx by bridging the gaps between genotype, gene expression, and disease. Nat Genet 49:1664–1670. doi:10.1038/ng.3969. 29019975 PMC6636856

[19] GTEx Consortium. 2015 Human genomics. The Genotype-Tissue Expression (GTEx) pilot analysis: multitissue gene regulation in humans. Science 348:648–660. doi:10.1126/science.1262110. 25954001 PMC4547484

[20] Gamazon ER, Wheeler HE, Shah KP, Mozaffari SV, Aquino-Michaels K, Carroll RJ, Eyler AE, Denny JC, GTEx Consortium, Nicolae DL, Cox NJ, Im HK 2015 A gene-based association method for mapping traits using reference transcriptome data. Nat Genet 47:1091–1098. doi:10.1038/ng.3367. 26258848 PMC4552594

[21] Barbeira A, Dickinson SP, Torres JM, Bonazzola R, Zheng J, Torstenson ES, Wheeler HE, Shah KP, Edwards T, Garcia T, GTEx Consortium, Nicolae D, Cox NJ, Im HK 2016 Integrating tissue specific mechanisms into GWAS summary results. BioRxiv doi:10.1101/045260.

[22] Di Mari JF, Mifflin RC, Powell DW 2005 The role of protein kinase C in gastrointestinal function and disease. Gastroenterology 128:2131–2146. doi:10.1053/j.gastro.2004.09.078. 15940644

[23] Meisel M, Hermann-Kleiter N, Hinterleitner R, Gruber T, Wachowicz K, Pfeifhofer-Obermair C, Fresser F, Leitges M, Soldani C, Viola A, Kaminski S, Baier G 2013 The kinase PKCα selectively upregulates interleukin-17A during Th17 cell immune responses. Immunity 38:41–52. doi:10.1016/j.immuni.2012.09.021. 23290522 PMC3556779

[24] Zhao GH, Fang YQ, Ryan U, Guo YX, Wu F, Du SZ, Chen DK, Lin Q 2016 Dynamics of Th17 associating cytokines in Cryptosporidium parvum-infected mice. Parasitol Res 115:879–887. doi:10.1007/s00436-015-4831-2. 26593737

[25] Clayburgh DR, Musch MW, Leitges M, Fu Y-X, Turner JR 2006 Coordinated epithelial NHE3 inhibition and barrier dysfunction are required for TNF-mediated diarrhea in vivo. J Clin Invest 116:2682–2694. doi:10.1172/JCI29218. 17016558 PMC1578628

[26] Sun A, Zhang H, Pang F, Niu G, Chen J, Chen F, Zhang J 2018 Essential genes of the macrophage response to Staphylococcus aureus exposure. Cell Mol Biol Lett 23:25. doi:10.1186/s11658-018-0090-4. 29849669 PMC5966896

[27] Wu Y, Zhang L, Zhang Y, Zhen Y, Liu S 2018 Bioinformatics analysis to screen for critical genes between survived and non‑survived patients with sepsis. Mol Med Rep 18:3737–3743. doi:10.3892/mmr.2018.9408. 30132542 PMC6131361

[28] Arenas AF, Salcedo GE, Gomez-Marin JE 2017 R script approach to infer toxoplasma infection mechanisms from microarrays and domain-domain protein interactions. Bioinform Biol Insights 11:1177932217747256. doi:10.1177/1177932217747256. 29317802 PMC5753922

[29] Assani K, Shrestha CL, Robledo-Avila F, Rajaram MV, Partida-Sanchez S, Schlesinger LS, Kopp BT 2017 Human cystic fibrosis macrophages have defective calcium-dependent protein kinase C activation of the NADPH oxidase, an effect augmented by Burkholderia cenocepacia. J Immunol 198:1985–1994. doi:10.4049/jimmunol.1502609. 28093527 PMC5322234

[30] Wang W, Wang Y, Debing Y, Zhou X, Yin Y, Xu L, Herrera Carrillo E, Brandsma JH, Poot RA, Berkhout B, Neyts J, Peppelenbosch MP, Pan Q 2017 Biological or pharmacological activation of protein kinase C alpha constrains hepatitis E virus replication. Antiviral Res 140:1–12. doi:10.1016/j.antiviral.2017.01.005. 28077314

[31] Chen X-M, O'Hara SP, Huang BQ, Splinter PL, Nelson JB, LaRusso NF 2005 Localized glucose and water influx facilitates Cryptosporidium parvum cellular invasion by means of modulation of host-cell membrane protrusion. Proc Natl Acad Sci U S A 102:6338–6343. doi:10.1073/pnas.0408563102. 15851691 PMC1088355

[32] Chen X-M, Splinter PL, Tietz PS, Huang BQ, Billadeau DD, LaRusso NF 2004 Phosphatidylinositol 3-kinase and frabin mediate Cryptosporidium parvum cellular invasion via activation of Cdc42. J Biol Chem 279:31671–31678. doi:10.1074/jbc.M401592200. 15133042

[33] Crane JK, Oh JS 1997 Activation of host cell protein kinase C by enteropathogenic Escherichia coli. Infect Immun 65:3277–3285. doi:10.1128/IAI.65.8.3277-3285.1997. 9234787 PMC175464

[34] Sukumaran SK, Prasadarao NV 2002 Regulation of protein kinase C in Escherichia coli K1 invasion of human brain microvascular endothelial cells. J Biol Chem 277:12253–12262. doi:10.1074/jbc.M110740200. 11805101

[35] Keys KL, Mak ACY, White MJ, Eckalbar WL, Dahl AW, Mefford J, Mikhaylova AV, Contreras MG, Elhawary JR, Eng C, Hu D, Hunstman S, Oh SS, Salazar S, Lenoir MA, Ye JC, Thornton TA, Zaitlen N, Burchard EG, Gignoux CR 2019 On the cross-population generalizability of gene expression prediction models. bioRxiv doi:10.1101/552042. PMC7449671 32797036

[36] Yang S-K, Hong M, Zhao W, Jung Y, Tayebi N, Ye BD, Kim K-J, Park SH, Lee I, Shin HD, Cheong HS, Kim LH, Kim H-J, Jung S-A, Kang D, Youn H-S, Liu J, Song K 2013 Genome-wide association study of ulcerative colitis in Koreans suggests extensive overlapping of genetic susceptibility with Caucasians. Inflamm Bowel Dis 19:954–966. doi:10.1097/MIB.0b013e3182802ab6. 23511034

[37] Genetics Consortium UI, Barrett JC, Lee JC, Lees CW, Prescott NJ, Anderson CA, Phillips A, Wesley E, Parnell K, Zhang H, Drummond H, Nimmo ER, Massey D, Blaszczyk K, Elliott T, Cotterill L, Dallal H, Lobo AJ, Mowat C, Sanderson JD, Jewell DP, Newman WG, Edwards C, Ahmad T, Mansfield JC, Satsangi J, Parkes M, Mathew CG, Wellcome Trust Case Control Consortium 2, Donnelly P, Peltonen L, Blackwell JM, Bramon E, Brown MA, Casas JP, Corvin A, Craddock N, Deloukas P, Duncanson A, Jankowski J, Markus HS, Mathew CG, McCarthy MI, Palmer CNA, Plomin R, Rautanen A, Sawcer SJ, Samani N, 2009 Genome-wide association study of ulcerative colitis identifies three new susceptibility loci, including the HNF4A region. Nat Genet 41:1330–1334. doi:10.1038/ng.483. 19915572 PMC2812019

[38] Ellinghaus D, Psoriasis Association Genetics Extension (PAGE), Jostins L, Spain SL, Cortes A, Bethune J, Han B, Park YR, Raychaudhuri S, Pouget JG, Hübenthal M, Folseraas T, Wang Y, Esko T, Metspalu A, Westra H-J, Franke L, Pers TH, Weersma RK, Collij V, D'Amato M, Halfvarson J, Jensen AB, Lieb W, Degenhardt F, Forstner AJ, Hofmann A, Schreiber S, Mrowietz U, Juran BD, Lazaridis KN, Brunak S, Dale AM, Trembath RC, Weidinger S, Weichenthal M, Ellinghaus E, Elder JT, Barker JNWN, Andreassen OA, McGovern DP, Karlsen TH, Barrett JC, Parkes M, Brown MA, Franke A 2016 Analysis of five chronic inflammatory diseases identifies 27 new associations and highlights disease-specific patterns at shared loci. Nat Genet 48:510–518. doi:10.1038/ng.3528. 26974007 PMC4848113

[39] Silverberg MS, Cho JH, Rioux JD, McGovern DPB, Wu J, Annese V, Achkar J-P, Goyette P, Scott R, Xu W, Barmada MM, Klei L, Daly MJ, Abraham C, Bayless TM, Bossa F, Griffiths AM, Ippoliti AF, Lahaie RG, Latiano A, Paré P, Proctor DD, Regueiro MD, Steinhart AH, Targan SR, Schumm LP, Kistner EO, Lee AT, Gregersen PK, Rotter JI, Brant SR, Taylor KD, Roeder K, Duerr RH 2009 Ulcerative colitis-risk loci on chromosomes 1p36 and 12q15 found by genome-wide association study. Nat Genet 41:216–220. doi:10.1038/ng.275. 19122664 PMC2652837

[40] Anderson CA, Boucher G, Lees CW, Franke A, D'Amato M, Taylor KD, Lee JC, Goyette P, Imielinski M, Latiano A, Lagacé C, Scott R, Amininejad L, Bumpstead S, Baidoo L, Baldassano RN, Barclay M, Bayless TM, Brand S, Büning C, Colombel J-F, Denson LA, De Vos M, Dubinsky M, Edwards C, Ellinghaus D, Fehrmann RSN, Floyd JAB, Florin T, Franchimont D, Franke L, Georges M, Glas J, Glazer NL, Guthery SL, Haritunians T, Hayward NK, Hugot J-P, Jobin G, Laukens D, Lawrance I, Lémann M, Levine A, Libioulle C, Louis E, McGovern DP, Milla M, Montgomery GW, Morley KI, Mowat C, 2011 Meta-analysis identifies 29 additional ulcerative colitis risk loci, increasing the number of confirmed associations to 47. Nat Genet 43:246–252. doi:10.1038/ng.764. 21297633 PMC3084597

[41] Franke A, Balschun T, Sina C, Ellinghaus D, Häsler R, Mayr G, Albrecht M, Wittig M, Buchert E, Nikolaus S, Gieger C, Wichmann HE, Sventoraityte J, Kupcinskas L, Onnie CM, Gazouli M, Anagnou NP, Strachan D, McArdle WL, Mathew CG, Rutgeerts P, Vermeire S, Vatn MH, IBSEN Study Group, Krawczak M, Rosenstiel P, Karlsen TH, Schreiber S 2010 Genome-wide association study for ulcerative colitis identifies risk loci at 7q22 and 22q13 (IL17REL). Nat Genet 42:292–294. doi:10.1038/ng.553. 20228798

[42] McGovern DPB, NIDDK IBD Genetics Consortium, Gardet A, Törkvist L, Goyette P, Essers J, Taylor KD, Neale BM, Ong RTH, Lagacé C, Li C, Green T, Stevens CR, Beauchamp C, Fleshner PR, Carlson M, D'Amato M, Halfvarson J, Hibberd ML, Lördal M, Padyukov L, Andriulli A, Colombo E, Latiano A, Palmieri O, Bernard E-J, Deslandres C, Hommes DW, de Jong DJ, Stokkers PC, Weersma RK, Sharma Y, Silverberg MS, Cho JH, Wu J, Roeder K, Brant SR, Schumm LP, Duerr RH, Dubinsky MC, Glazer NL, Haritunians T, Ippoliti A, Melmed GY, Siscovick DS, Vasiliauskas EA, Targan SR, Annese V, Wijmenga C, 2010 Genome-wide association identifies multiple ulcerative colitis susceptibility loci. Nat Genet 42:332–337. doi:10.1038/ng.549. 20228799 PMC3087600

[43] Jostins L, International IBD Genetics Consortium (IIBDGC), Ripke S, Weersma RK, Duerr RH, McGovern DP, Hui KY, Lee JC, Schumm LP, Sharma Y, Anderson CA, Essers J, Mitrovic M, Ning K, Cleynen I, Theatre E, Spain SL, Raychaudhuri S, Goyette P, Wei Z, Abraham C, Achkar J-P, Ahmad T, Amininejad L, Ananthakrishnan AN, Andersen V, Andrews JM, Baidoo L, Balschun T, Bampton PA, Bitton A, Boucher G, Brand S, Büning C, Cohain A, Cichon S, D'Amato M, De Jong D, Devaney KL, Dubinsky M, Edwards C, Ellinghaus D, Ferguson LR, Franchimont D, Fransen K, Gearry R, Georges M, Gieger C, 2012 Host-microbe interactions have shaped the genetic architecture of inflammatory bowel disease. Nature 491:119–124. doi:10.1038/nature11582. 23128233 PMC3491803

[44] de Lange KM, Moutsianas L, Lee JC, Lamb CA, Luo Y, Kennedy NA, Jostins L, Rice DL, Gutierrez-Achury J, Ji S-G, Heap G, Nimmo ER, Edwards C, Henderson P, Mowat C, Sanderson J, Satsangi J, Simmons A, Wilson DC, Tremelling M, Hart A, Mathew CG, Newman WG, Parkes M, Lees CW, Uhlig H, Hawkey C, Prescott NJ, Ahmad T, Mansfield JC, Anderson CA, Barrett JC 2017 Genome-wide association study implicates immune activation of multiple integrin genes in inflammatory bowel disease. Nat Genet 49:256–261. doi:10.1038/ng.3760. 28067908 PMC5289481

[45] Wojcik GL, Marie C, Abhyankar MM, Yoshida N, Watanabe K, Mentzer AJ, Carstensen T, Mychaleckyj J, Kirkpatrick BD, Rich SS, Concannon P, Haque R, Tsokos GC, Petri WA, Duggal P, Wojcik GL, Marie C, Abhyankar MM, Yoshida N, Watanabe K, Mentzer AJ, Carstensen T, Mychaleckyj J, Kirkpatrick BD, Rich SS, Concannon P, Haque R, Tsokos GC, Petri WA, Duggal P 2018 Genome-wide association study reveals genetic link between diarrhea-associated Entamoeba histolytica infection and inflammatory bowel disease. mBio 9:e01668-18. doi:10.1128/mBio.01668-18. 30228239 PMC6143743

[46] Liu T-L, Fan X-C, Li Y-H, Yuan Y-J, Yin Y-L, Wang X-T, Zhang L-X, Zhao G-H 2018 Expression profiles of mRNA and lncRNA in HCT-8 cells infected with Cryptosporidium parvum IId subtype. Front Microbiol 9:1409. doi:10.3389/fmicb.2018.01409. 30013528 PMC6036261

[47] Baier G, Wagner J 2009 PKC inhibitors: potential in T cell-dependent immune diseases. Curr Opin Cell Biol 21:262–267. doi:10.1016/j.ceb.2008.12.008. 19195860

[48] Ruuska T, Vesikari T 1990 Rotavirus disease in Finnish children: use of numerical scores for clinical severity of diarrhoeal episodes. Scand J Infect Dis 22:259–267. doi:10.3109/00365549009027046. 2371542

[49] Liu J, Gratz J, Amour C, Kibiki G, Becker S, Janaki L, Verweij JJ, Taniuchi M, Sobuz SU, Haque R, Haverstick DM, Houpt ER 2013 A laboratory-developed TaqMan array card for simultaneous detection of 19 enteropathogens. J Clin Microbiol 51:472–480. doi:10.1128/JCM.02658-12. 23175269 PMC3553916

[50] Delaneau O, Marchini J, Zagury J-F 2011 A linear complexity phasing method for thousands of genomes. Nat Methods 9:179–181. doi:10.1038/nmeth.1785. 22138821

[51] Marchini J, Howie B, Myers S, McVean G, Donnelly P 2007 A new multipoint method for genome-wide association studies by imputation of genotypes. Nat Genet 39:906–913. doi:10.1038/ng2088. 17572673

[52] Howie BN, Donnelly P, Marchini J 2009 A flexible and accurate genotype imputation method for the next generation of genome-wide association studies. PLoS Genet 5:e1000529. doi:10.1371/journal.pgen.1000529. 19543373 PMC2689936

[53] Wellcome Trust Case Control Consortium. 2007 Genome-wide association study of 14,000 cases of seven common diseases and 3,000 shared controls. Nature 447:661–678. doi:10.1038/nature05911. 17554300 PMC2719288

[54] Marchini J, Howie B 2010 Genotype imputation for genome-wide association studies. Nat Rev Genet 11:499–511. doi:10.1038/nrg2796. 20517342

[55] Zhu S, Qian T, Hoshida Y, Shen Y, Yu J, Hao K 2019 GIGSEA: genotype imputed gene set enrichment analysis using GWAS summary level data. Bioinformatics 35:160–163. doi:10.1093/bioinformatics/bty529. 30010968 PMC6298047

[56] Liberzon A, Birger C, Thorvaldsdóttir H, Ghandi M, Mesirov JP, Tamayo P 2015 The Molecular Signatures Database (MSigDB) hallmark gene set collection. Cell Syst 1:417–425. doi:10.1016/j.cels.2015.12.004. 26771021 PMC4707969

[57] Nishimura D 2001 BioCarta. Biotech Software & Internet Report 2:117–120. doi:10.1089/152791601750294344.

[58] Subramanian A, Tamayo P, Mootha VK, Mukherjee S, Ebert BL, Gillette MA, Paulovich A, Pomeroy SL, Golub TR, Lander ES, Mesirov JP 2005 Gene set enrichment analysis: a knowledge-based approach for interpreting genome-wide expression profiles. Proc Natl Acad Sci U S A 102:15545–15550. doi:10.1073/pnas.0506580102. 16199517 PMC1239896

