# Supplementary figures and images for: Genome-Wide Association Study of Cryptosporidiosis in Infants Implicates PRKCA
Source: mBio. 2020 Feb 4;11(1):e03343-19. doi: 10.1128/mBio.03343-19 (PMC7002356; doi:10.1128/mBio.03343-19)

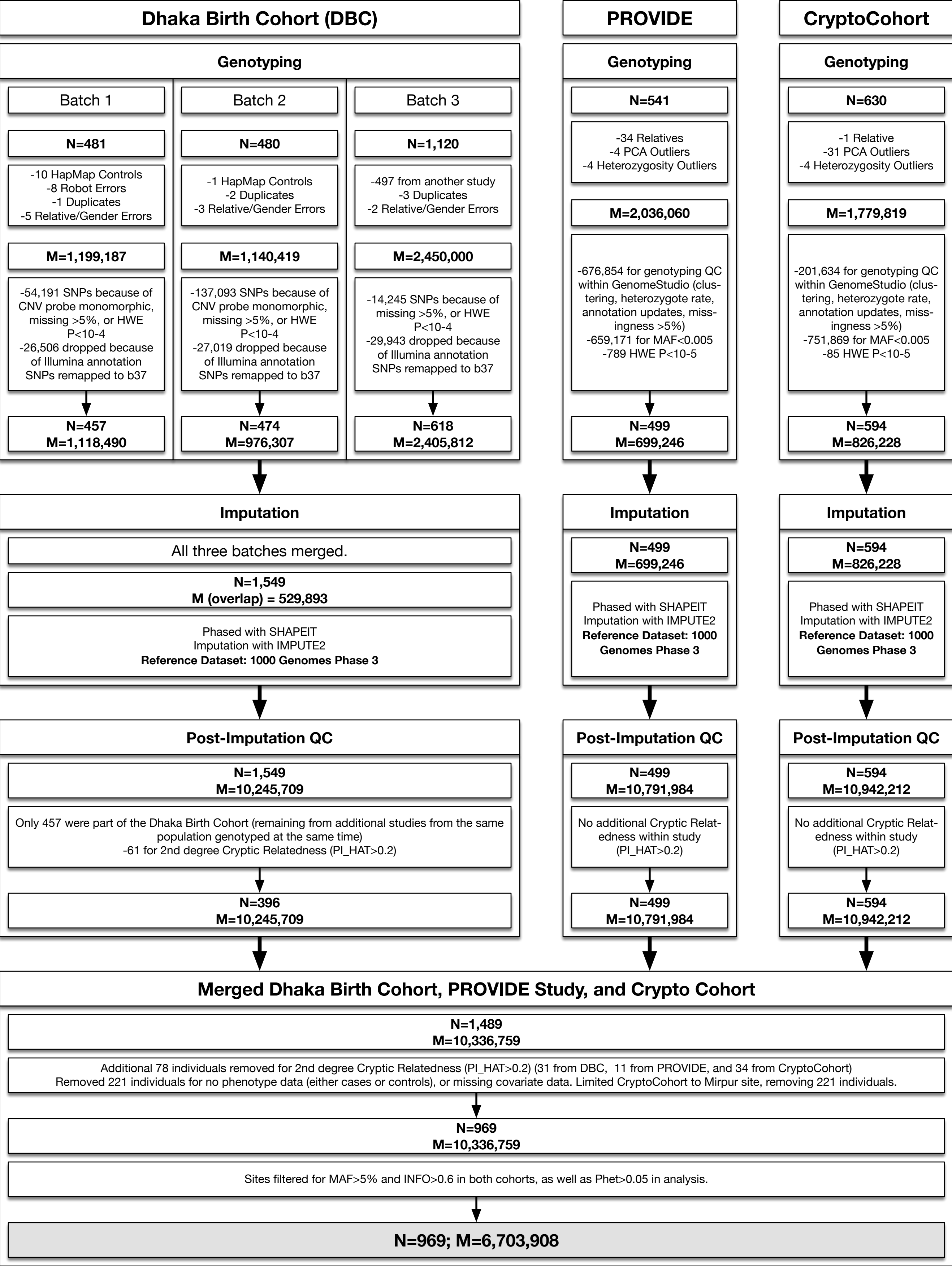

Supplement: FIG S1 [file mBio.03343-19-sf001.pdf]

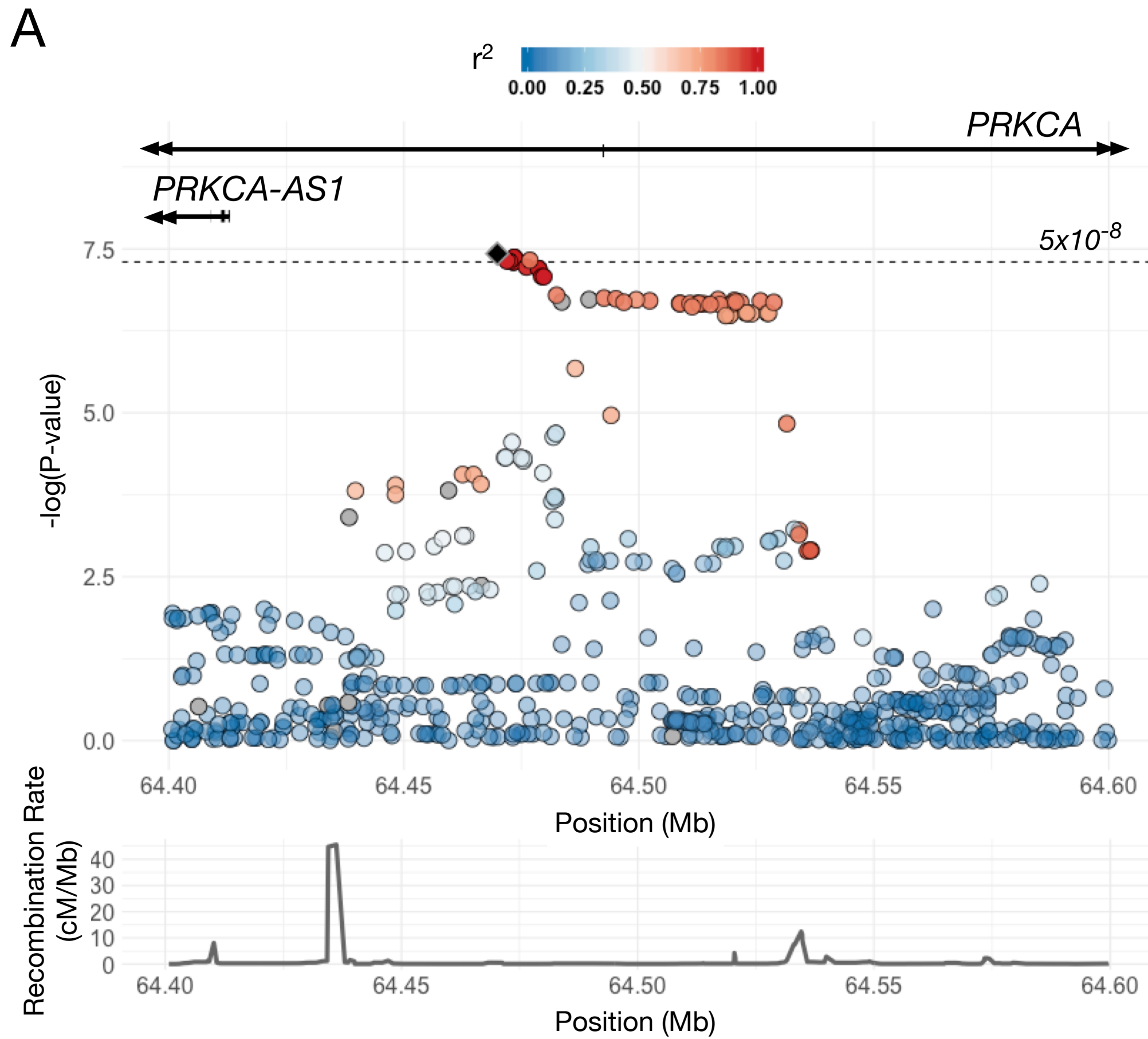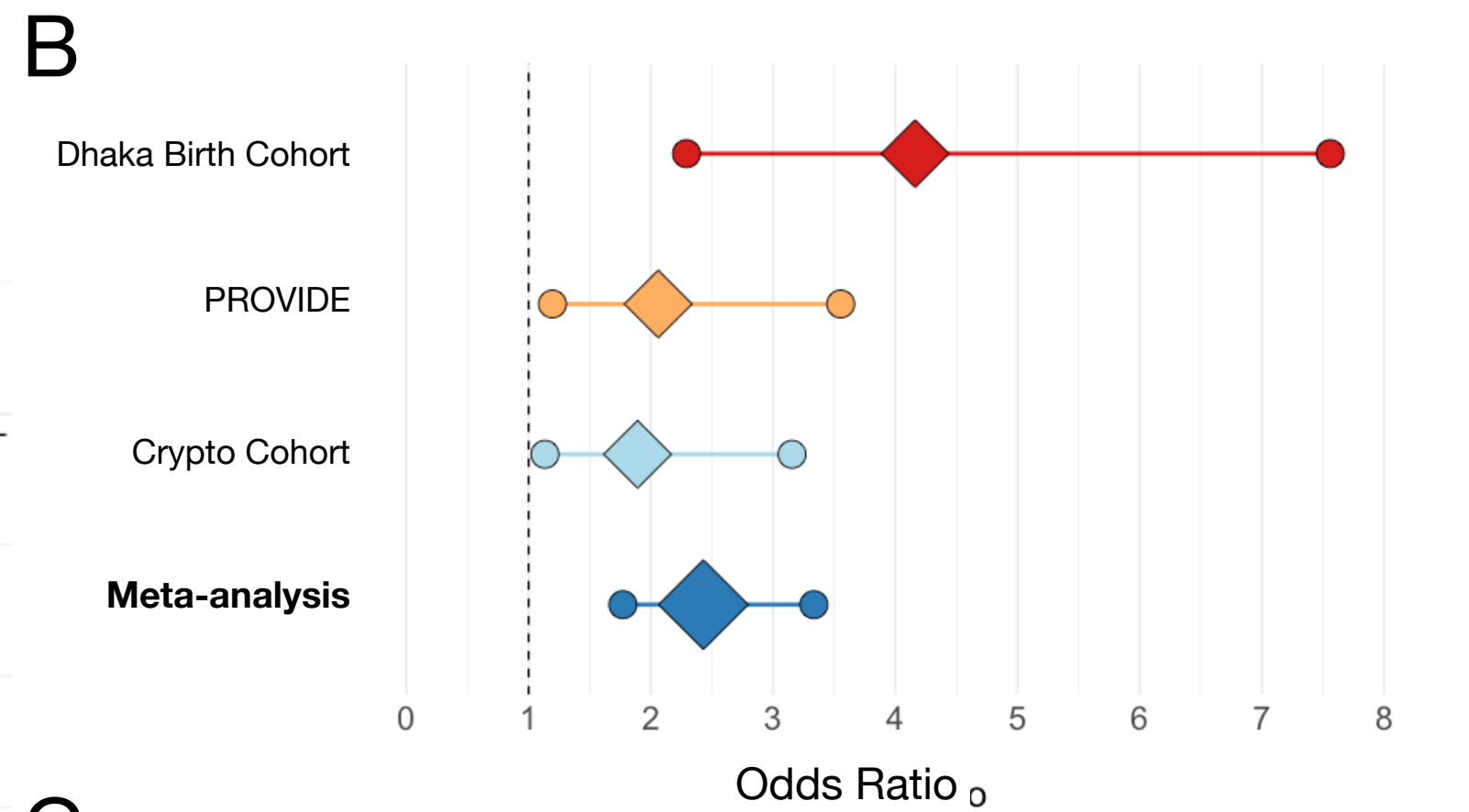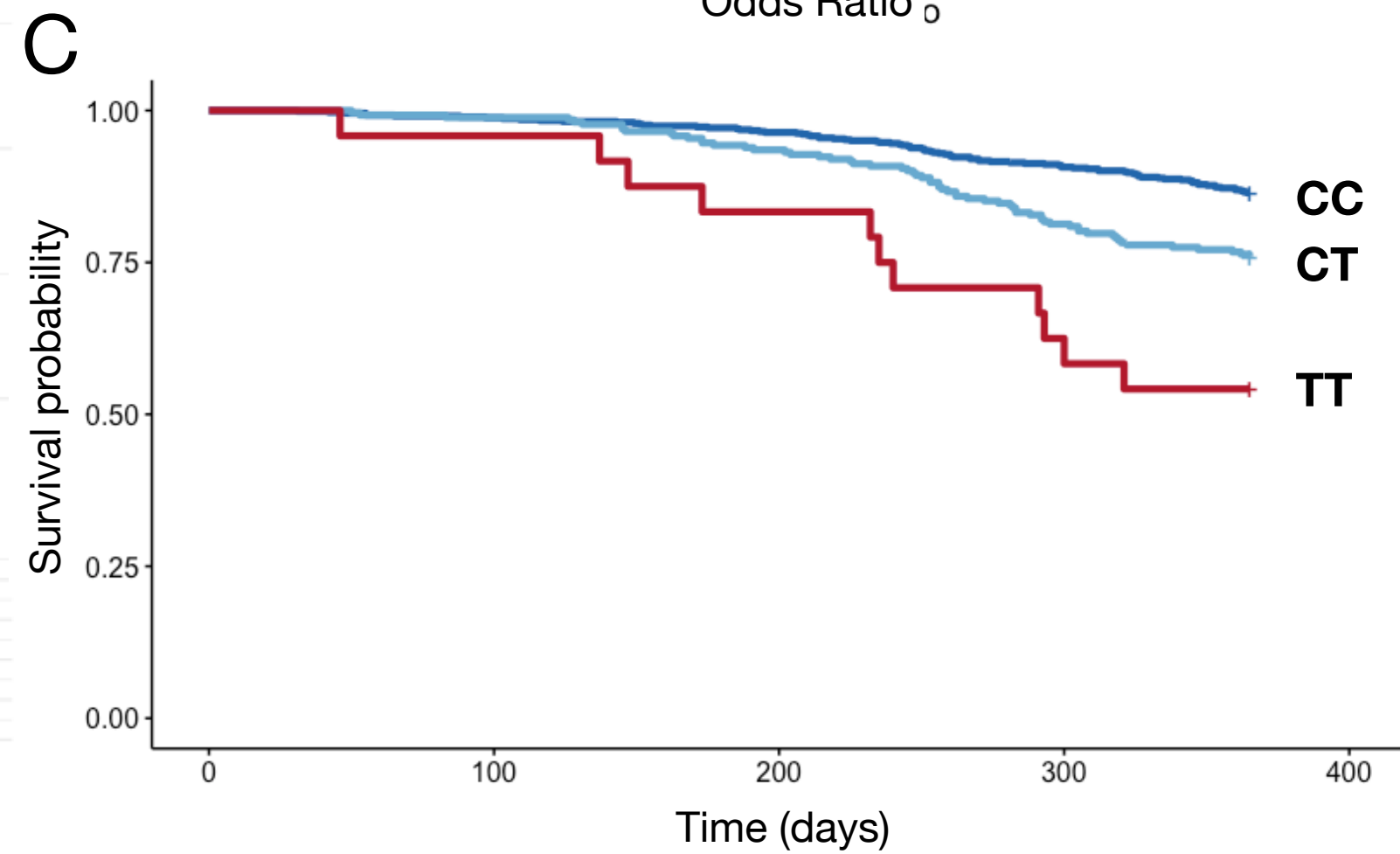

Supplement: FIG S2 [file mBio.03343-19-sf002.pdf]

**A**

Plotted SNPs

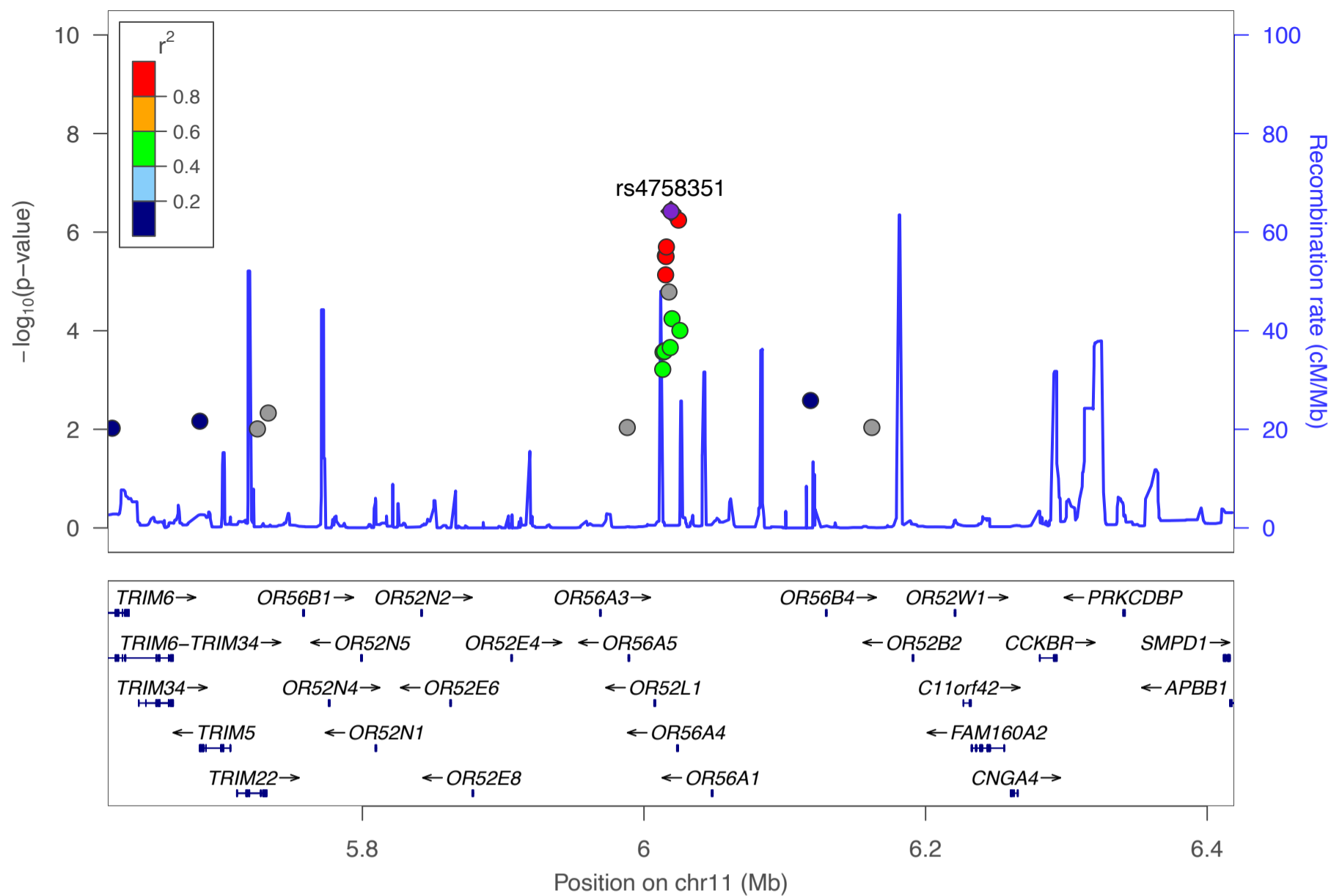**B**

Plotted SNPs

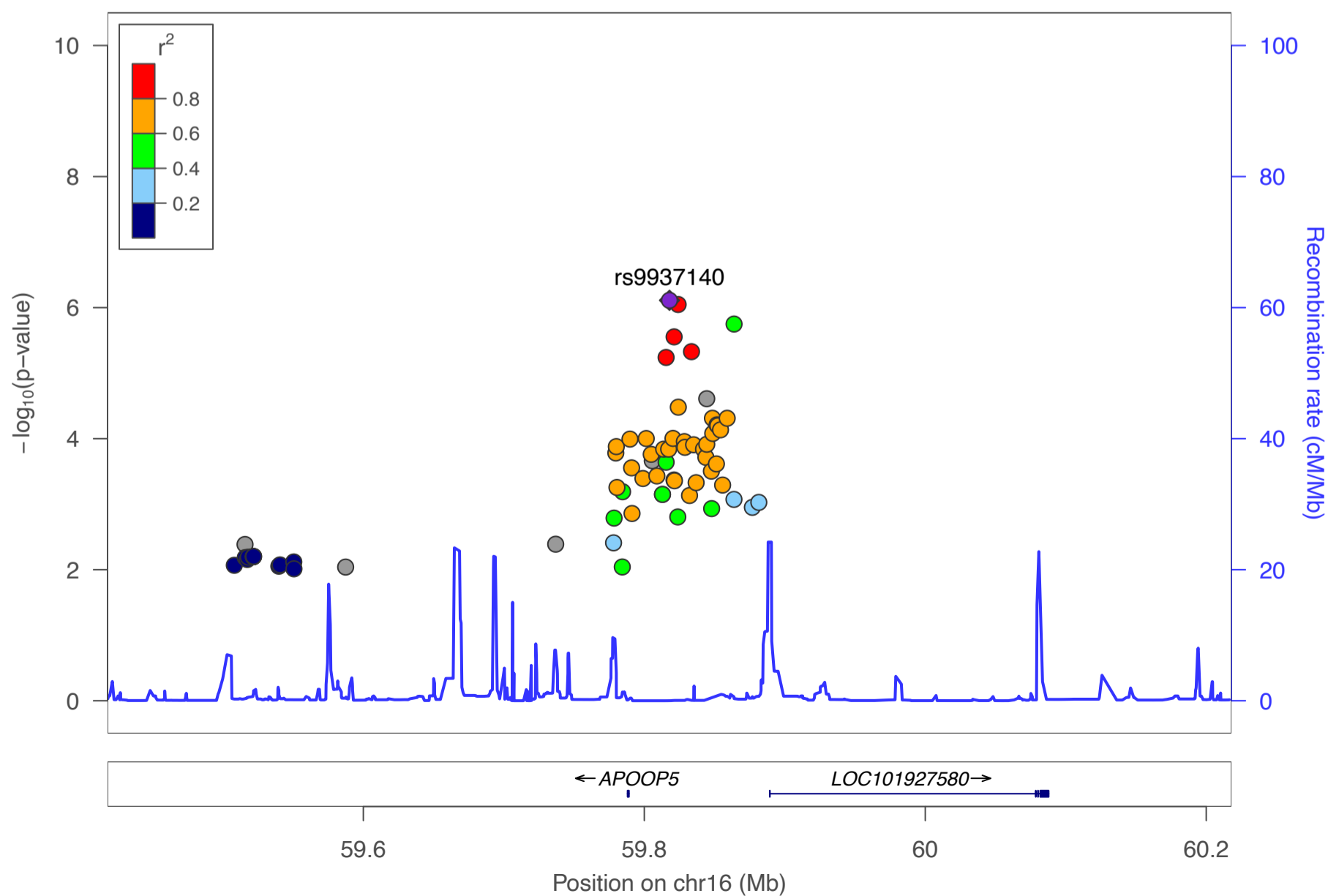

Supplement: FIG S3 [file mBio.03343-19-sf003.pdf]

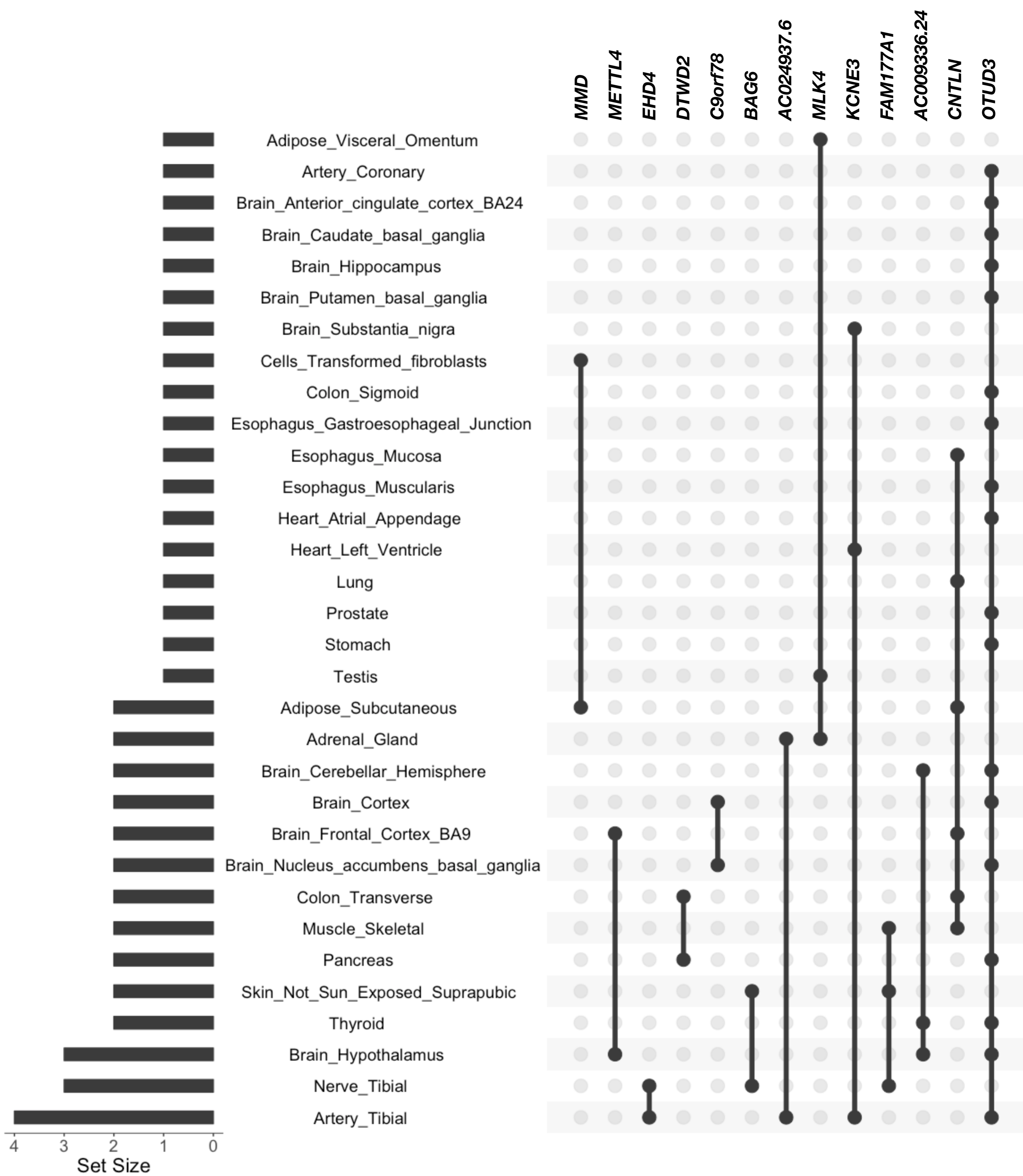

Supplement: FIG S4 [file mBio.03343-19-sf004.pdf]

**A**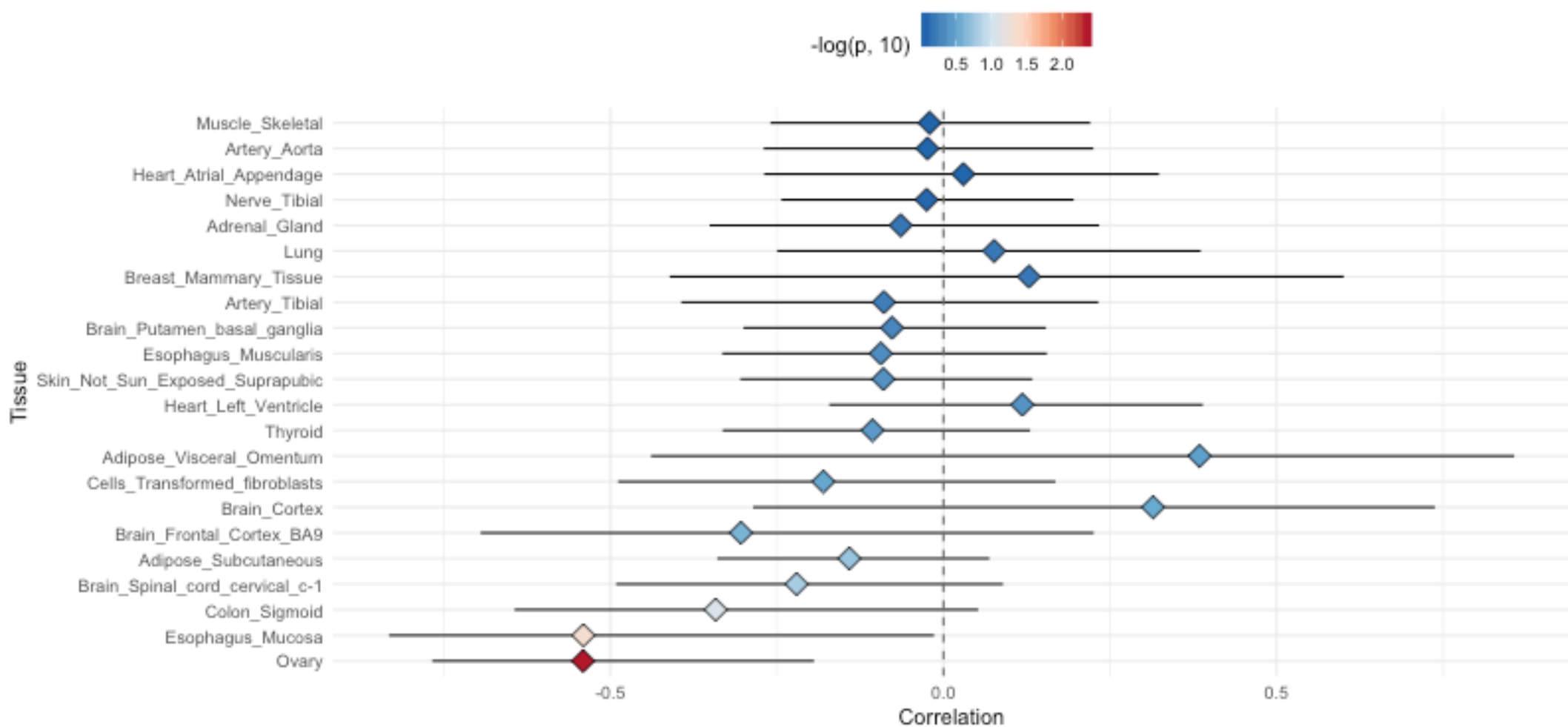**B**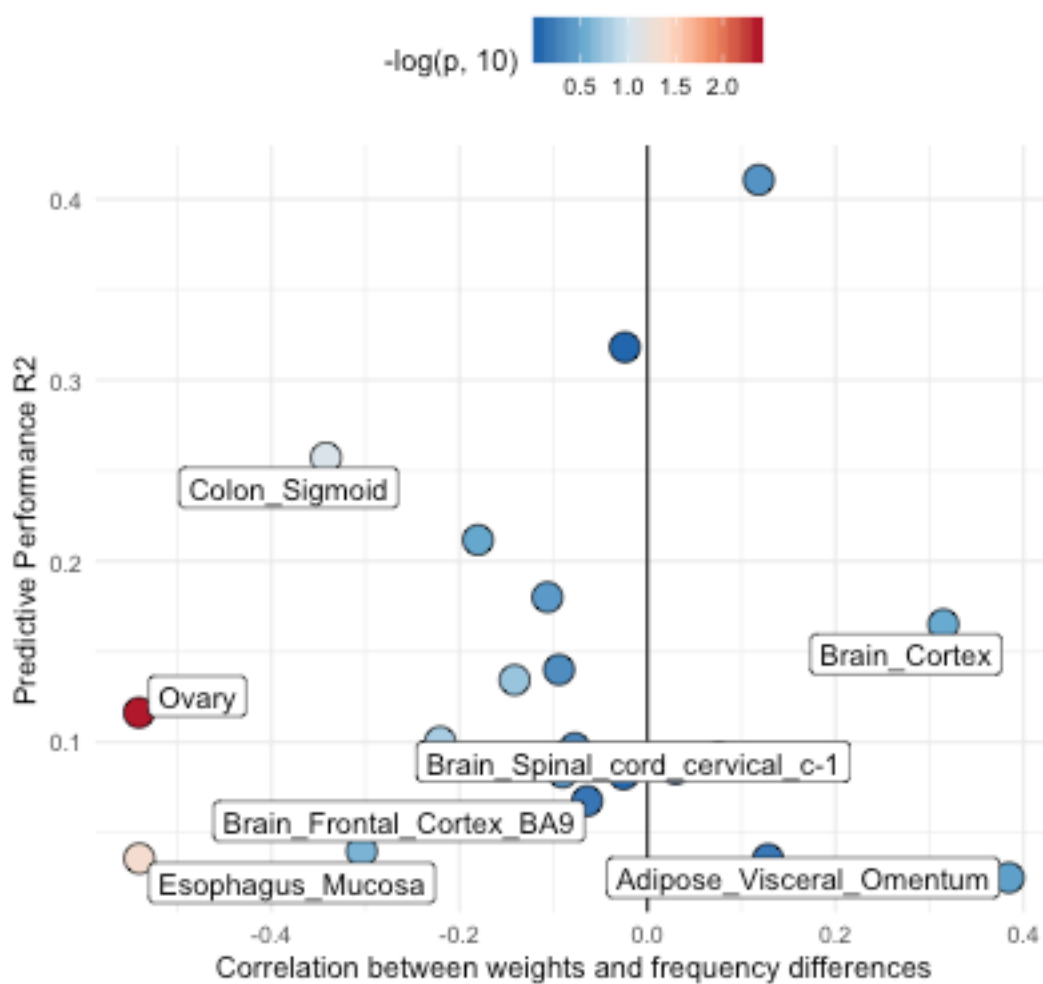**C**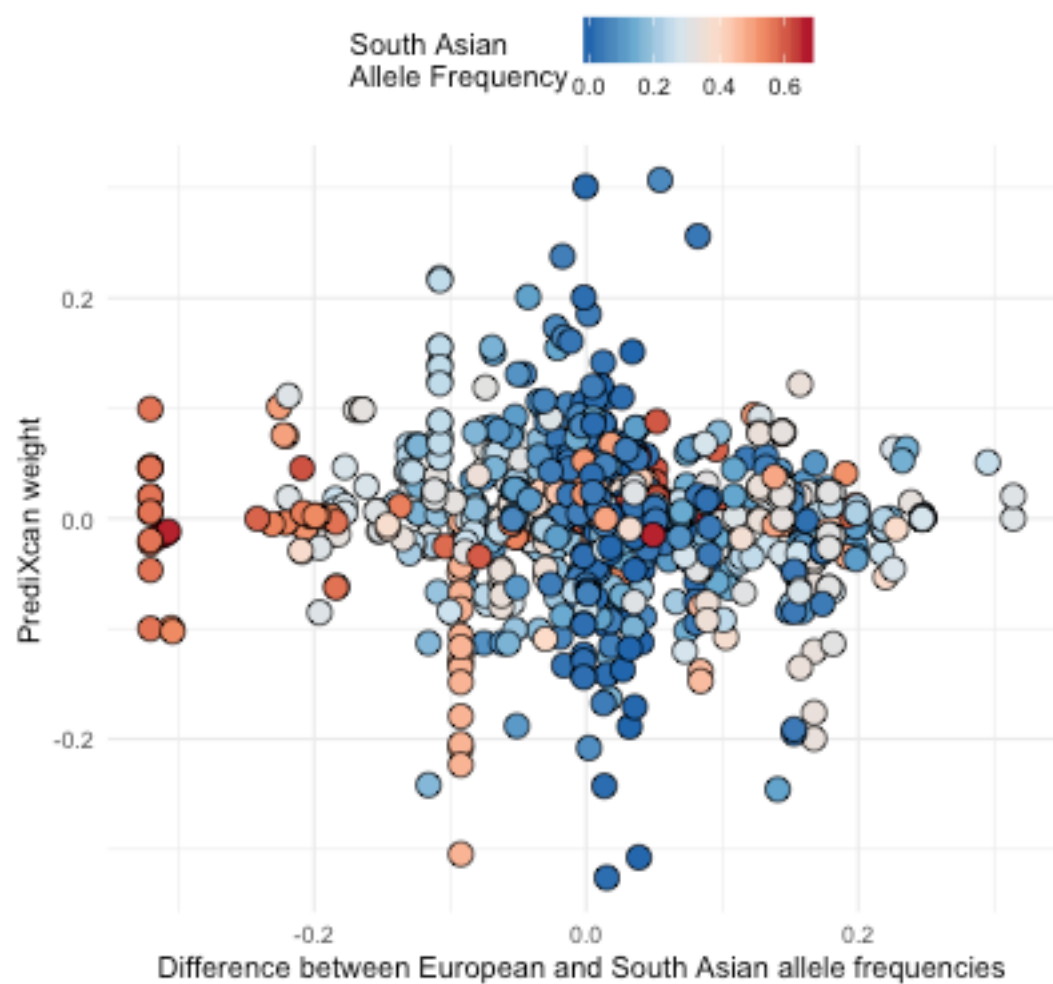

Supplement: FIG S5 [file mBio.03343-19-sf005.pdf]

A

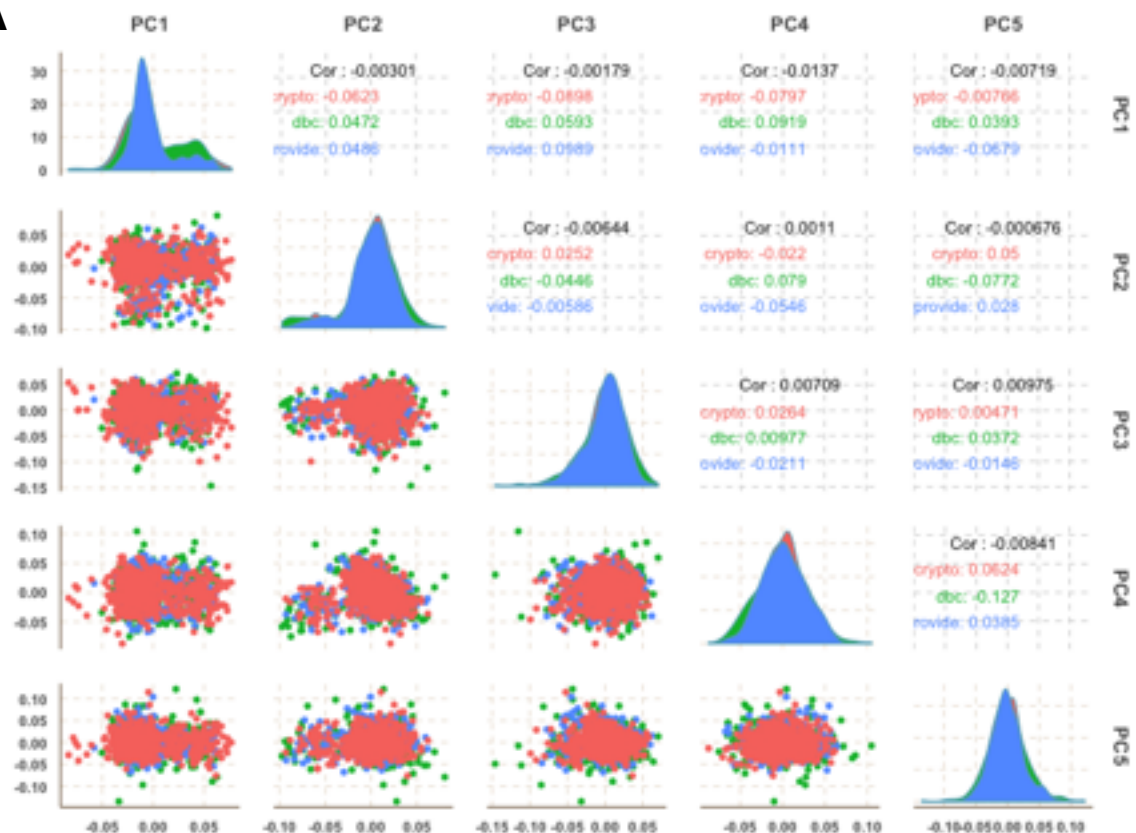

B

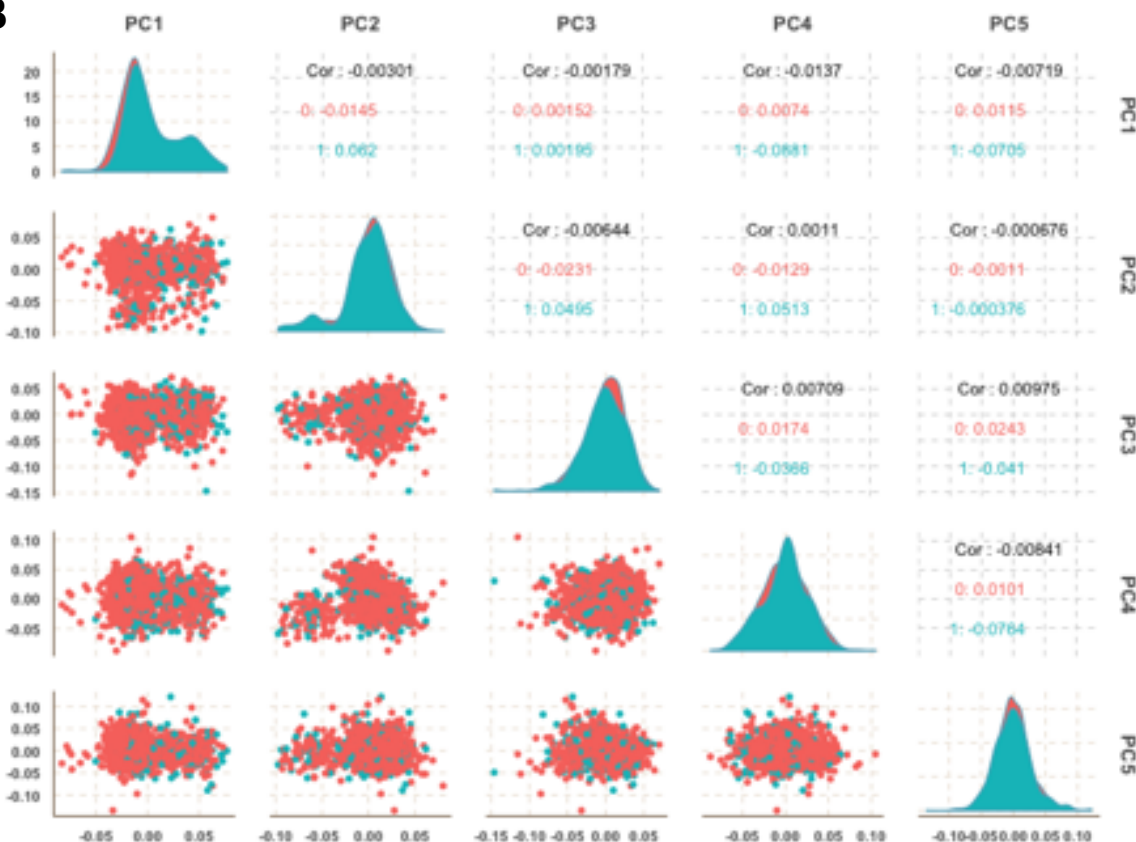

C

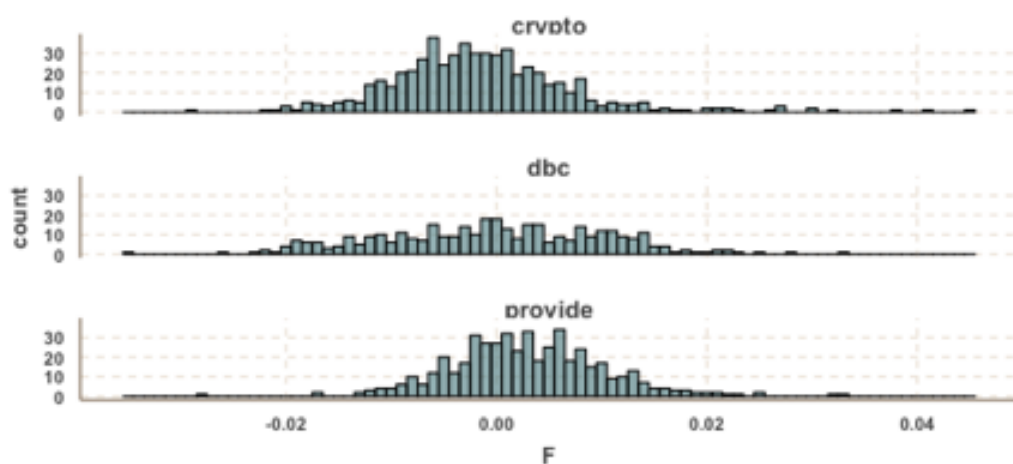

Supplement: FIG S6 [file mBio.03343-19-sf006.pdf]

**A**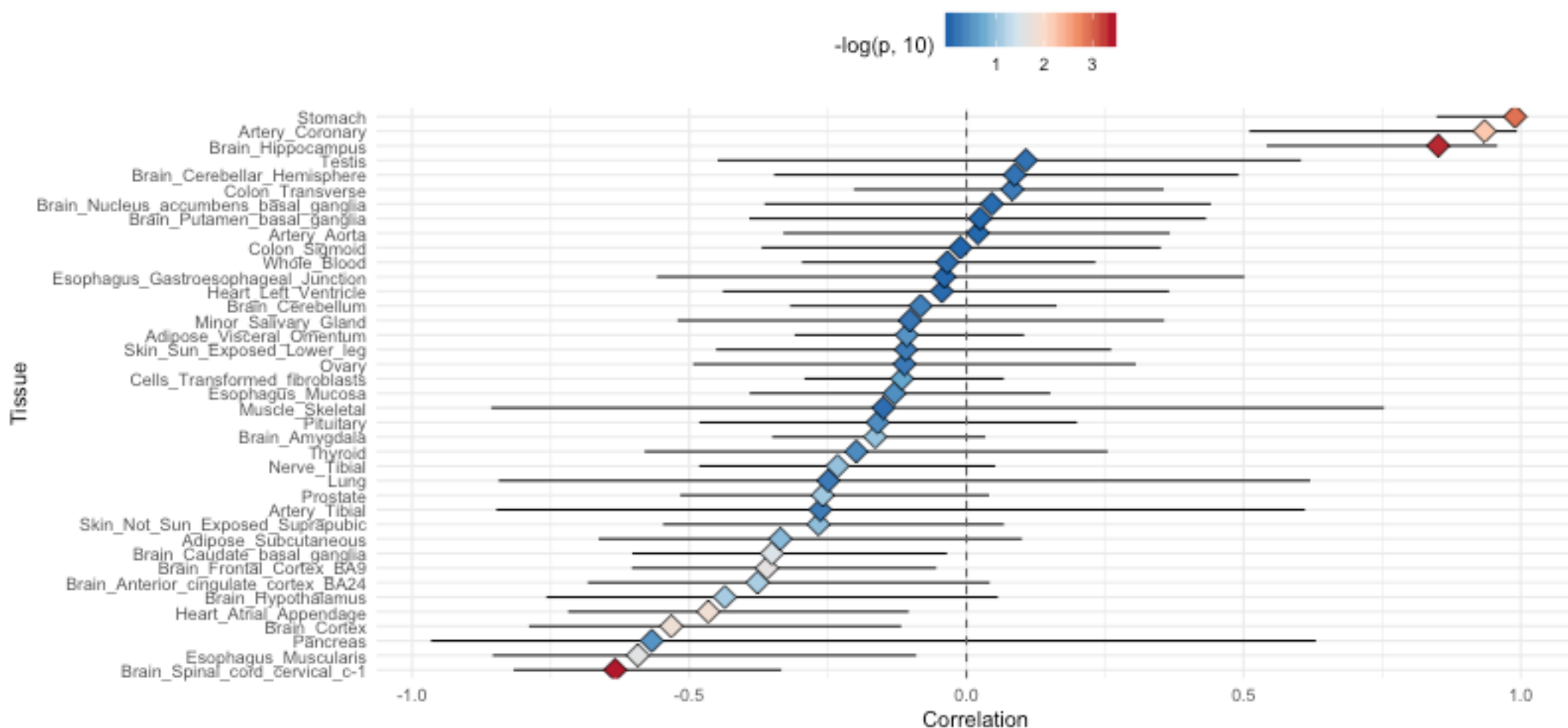**B**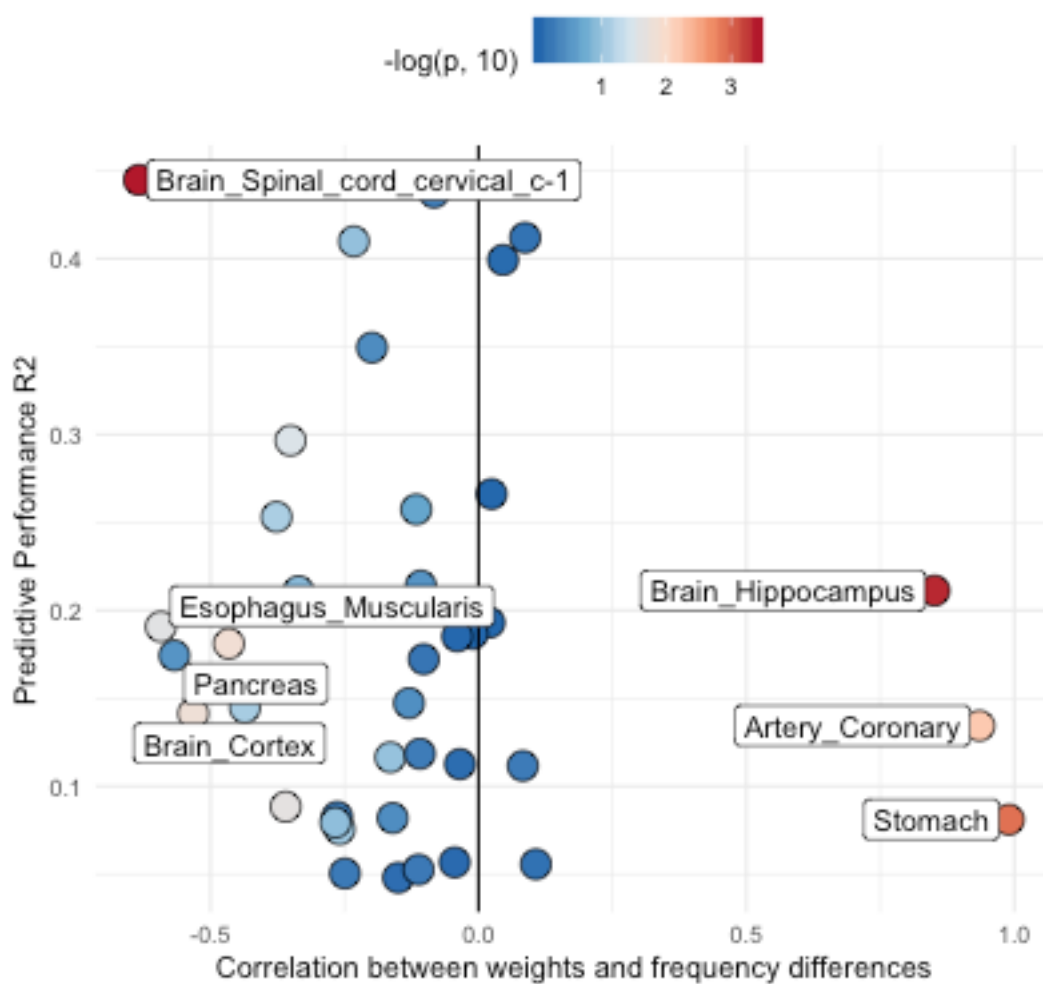**C**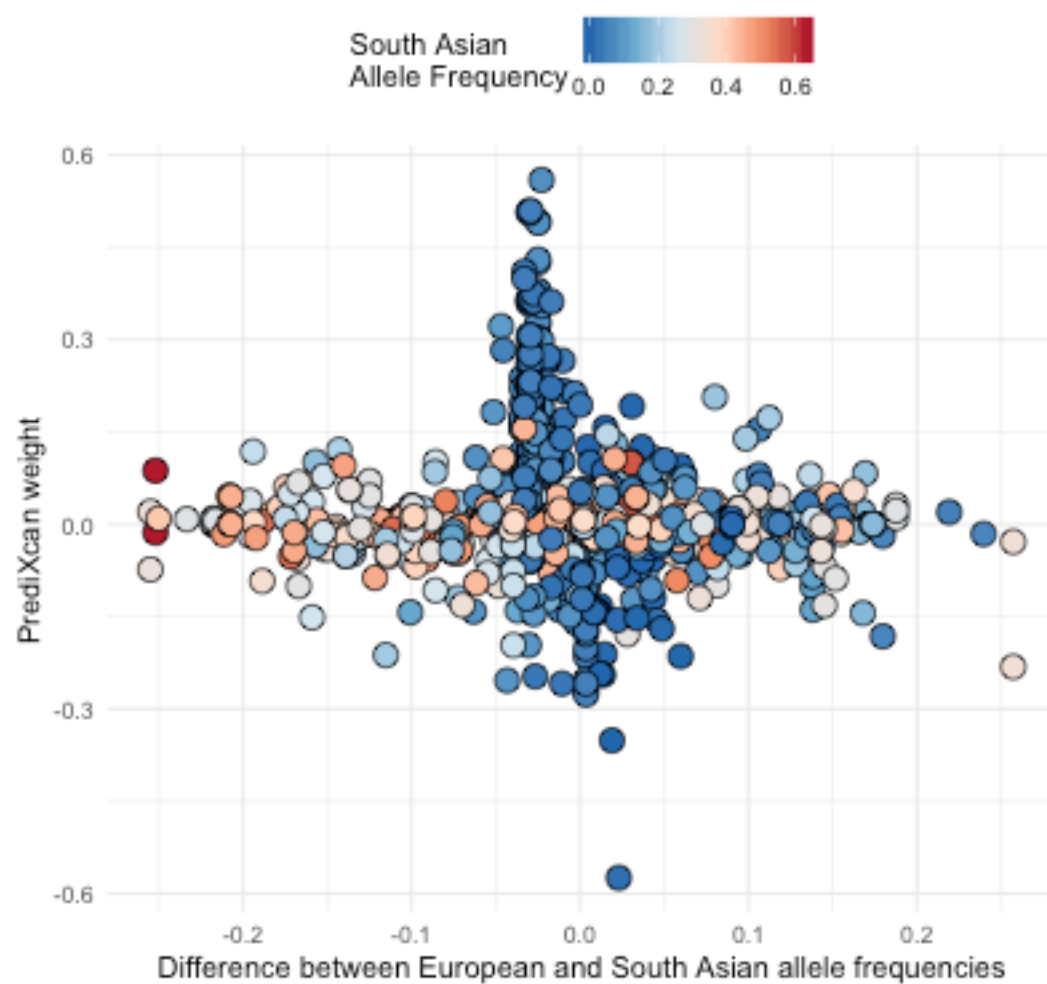

Supplement: FIG S7 [file mBio.03343-19-sf007.pdf]
